# MAVSCOT: A fuzzy logic-based HIV diagnostic system with indigenous multi-lingual interfaces for rural Africa

**DOI:** 10.1371/journal.pone.0241864

**Published:** 2020-11-06

**Authors:** Olugbenga Oluseun Oluwagbemi, Folakemi Etseoghena Oluwagbemi, Abdulwahab Jatto, Cang Hui

**Affiliations:** 1 Department of Mathematical Sciences, Stellenbosch University, Matieland, Stellenbosch, South Africa; 2 Department of Computer Science and Information Technology, Sol Plaatje University, Kimberley, South Africa; 3 Nuffield Department of Medicine, Big Data Institute, University of Oxford, Oxford, United Kingdom; 4 DST/NRF Centre of Excellence in Epidemiological Modelling and Analysis, Stellenbosch University, Stellenbosch, South Africa; 5 Hagerhof, Andringa and Borched Street, Stellenbosch, South Africa; 6 Department of Computer Science, Federal University Lokoja, Lokoja, Nigeria; 7 African Institute for Mathematical Sciences, Cape Town, South Africa; University of Defence, SERBIA

## Abstract

HIV still constitutes a major public health problem in Africa, where the highest incidence and prevalence of the disease can be found in many rural areas, with multiple indigenous languages being used for communication by locals. In many rural areas of the KwaZulu-Natal (KZN) in South Africa, for instance, the most widely used languages include Zulu and Xhosa, with only limited comprehension in English and Afrikaans. Health care practitioners for HIV diagnosis and treatment, often, cannot communicate efficiently with their indigenous ethnic patients. An informatics tool is urgently needed to facilitate these health care professionals for better communication with their patients during HIV diagnosis. Here, we apply fuzzy logic and speech technology and develop a fuzzy logic HIV diagnostic system with indigenous multi-lingual interfaces, named Multi-linguAl HIV indigenouS fuzzy logiC**-**based diagnOstic sysTem (MAVSCOT). This HIV multilingual informatics software can facilitate the diagnosis in underprivileged rural African communities. We provide examples on how MAVSCOT can be applied towards HIV diagnosis by using existing data from the literature. Compared to other similar tools, MAVSCOT can perform better due to its implementation of the fuzzy logic. We hope MAVSCOT would help health care practitioners working in indigenous communities of many African countries, to efficiently diagnose HIV and ultimately control its transmission.

## 1 Introduction

HIV is one of the diseases currently affecting many sub-Saharan African countries. Out of the 36.7 million people living with HIV globally, sub-Saharan Africa has an estimated 25.6 million, accounting for two-thirds of the global total [1]. To date, it has been estimated that only 60% of people living with HIV know their status [2]. This is disturbing, as the remaining 40% have not been able to access proper HIV testing services. Access to HIV diagnostic and testing services is not readily available to many rural and sub-urban dwellers. For instance, KwaZulu-Natal province in South Africa, has been known for its high incidence and prevalence of HIV [3–6] and the region is also known for low uptake of HIV prevention services [7]. Precise and efficient diagnosis of any disease, coupled with the appropriate treatments, will result in proper control [8]. Lack of access to HIV testing kits, lack of access to Antiretroviral Therapy (ART), amongst others, constitute great barriers.

Some issues of concern in the fight against the disease include: ignorance about the factors responsible for its transmission, illiteracy, and diversities in indigenous languages among residents of African countries. The latter issue has resulted in delays in HIV diagnosis and care provision, false pre-clinical diagnosis, and prescription among inhabitants of local and sub-urban communities [9–12]. In South Africa specifically, there are several indigenous languages such as Zulu, Xhosa, Setswana, Tswana, Venda, amongst others, with Zulu and Xhosa as the most widely used languages in many rural areas [13–15]. In the absence of interpreters and HIV test kits, due to limited resources, English-speaking medical doctors, nurses, and foreign medical personnel, often find it difficult to efficiently communicate in Zulu and Xhosa with indigenous ethnic patients at rural clinics for HIV diagnosis [11, 12, 16]. Such language barriers have the potential to cause frustration, miscommunication, time-wastage and in extreme cases, false diagnoses, and treatments. Consequently, we ask how health care practitioners can be supported to achieve effective HIV diagnosis and treatments especially among indigenous communities and sub-urban communities in Africa, and what computational methods can be implemented to complement the efforts of English-speaking medical doctors in diagnosing patients of indigenous local African communities?

The aim here is to develop an African indigenous multi-lingual HIV informatics system to aid effective and efficient diagnoses of HIV, and assist with the staging of HIV and the provision of recommendations for medical personnel attending to indigenous inhabitants of African communities in rural/sub-urban settings. The motivation to build this fuzzy system stems from the fact that existing systems developed so far lack the capacity to address the needs of medical doctors and medical personnel managing HIV treatments in indigenous rural communities of Africa, especially in local indigenous languages. We address some of the shortcomings that the language barrier has created between health workers and the patients among indigenous local African communities.

In particular, the developed HIV multi-lingual informatics system tool is to complement the efforts of medical personnel in such communities where efficient interpreters are often lacking. The software was developed in English language but made available for some indigenous South African languages such as Afrikaans, Zulu and Xhosa.

Here we introduce this informatics system in two aspects: (i) to explain the applied fuzzy logic and speech technology in the development of the Multi-linguAl HIV indigenouS fuzzy logiC-based DiagnOstic sysTem ((MAVSCOT), henceforth) in English, Afrikaans, Zulu and Xhosa, in order to facilitate HIV diagnosis in underprivileged rural communities in Africa; and (ii) to compare MAVSCOT with other related existing systems.

Fuzzy Sets was used in the system development because it can handle uncertainties associated with medical diagnosis. On a general context, the development and application of Fuzzy approaches have been extended to other research areas such as: applied sciences, from subsurface oil/gas assessment to disease diagnosis [17–22].

More recent literature have focused the application of fuzzy set theory and its generalizations on handling uncertainty in different domains which include: intelligent selection of supplier of medical consumption-related products [23], determination of school enrollment for students [24], aggregating expert opinions on the effective finding of Top Event (TE) in industrial processes [25], applied medical diagnosis [26], resolving uncertainties in medical prognosis and diagnosis [27], improving medical diagnosis [28], forecasting of surface roughness [29], and resolving uncertainties in medical diagnosis [30–32].

There have been previous works on developing systems for HIV diagnosis but each with its own limitations. Pazzani et al. [33] applied the knowledge of rule-based expert systems to the management of HIV-infected patients. Their system encoded information from existing literature of known drug resistant mutations. One of the limitations of their system was an incomplete understanding of the relationship between genomic mutations that confers drug resistance and the surrogate outcomes. Atalay et al. [34] developed an interactive web-based HIV patient care expert systems. They adopted a methodology that utilized HTML and CGI-script for implementation. Seidenberg et al. [35] developed a mobile-SMS-based system for early infant diagnosis of HIV infection in Zambia, which used mobile phone texting of blood test results to HIV screening laboratories. However, this system could not recommend the correct dosage of HIV anti-retroviral drugs, nor the expiration of such drugs. Ebrahimi et al. [36] developed an HIV/AIDS web-based medical consulting system that provides consulting services based on systematic input data. Their system, however, lacked multilingual features, thus unable to support foreign medical personnel working in rural African communities. Tucker et al. [37], examined how a telephone-based IVR (Interactive Voice Response) self-monitoring system, can be used to assess daily HIV anti-retroviral medication adherence. Their system lacked multilingual features and does not validate the expiry date of the HIV anti-retroviral medication. Having considered all these HIV-related systems, we highlight limitations associated with these existing systems: one of common limitations is that they are not multi-lingual. The existing systems so far lack the capacity to address the needs of managing HIV patients in indigenous rural communities in Africa, especially in local indigenous languages.

Here, we present a multi-lingual HIV indigenous fuzzy logic-based diagnostic system (MAVSCOT). The novelty of our work lies in the fact that our work is probably the first HIV diagnosis software available and proficient in multiple South African languages. This makes it useful in the hands of medical doctors, who undertake HIV medical service tasks among/within indigenous African communities.

## 2 Materials and methods

### 2.1 MAVSCOT system data

Data gathering was conducted by extensive survey of literature. Criteria used for the literature search and for the inclusion of HIV data include targeting research articles published between 2001 and 2016 that contain symptomatic data about People Living With HIV (PLWHIV), with specific search phrases “HIV”, “HIV Symptoms”, “commonly reported symptoms of HIV”, and “Symptoms”. See S1 File (the online supplementary file) and S1 Table for details.

A total of 66 HIV symptoms were included in the MAVSCOT software, with 29 HIV symptoms included for male patients and 39 for female patients. Some of the HIV symptoms incorporated into MAVSCOT software are: weight loss, poor sleep, muscle aches/joint pain, fatigue, anxiety/nervous, headache, memory loss, cough, shortness of breath, fever/chills, sweats, dizzy/dizziness, abdominal pain, diarrhea, loss of appetite, and rash [38–42]. There are three stages of HIV namely: the acute HIV infection stage, chronic HIV infection stage and advanced HIV stage (full blown AIDS). Information about symptoms associated with these HIV stages were also sourced from existing literature [43]. See the online supplementary file, S1 and S2 Tables for details

Information about the translation of the English language symptoms and texts of different sections within MAVSCOT, to Afrikaans, Zulu and IsiXhosa, were obtained from online translators such as Google Translate [44] and Microsoft Translator [45]. Anonymous indigenous speakers were also invited to verify the correct spellings and pronunciations of translations of HIV symptoms from English to indigenous languages. The compiled information from online translators and indigenous speakers of these South African languages can be found in S1 Appendix.

### 2.2 MAVSCOT architecture

MAVSCOT was developed to accept symptoms, diagnose, and predict the possible presence of HIV in an individual, based on HIV symptoms provided (S1 Fig). The system also provides advice to individuals. Predictability within the system is based on the symptoms keyed into the system and other factors. These other factors include social drivers of HIV such as lifestyle and knowledge of individuals about HIV mode of transmission [46–55]. The knowledge base and the inference engine form the two major components of the system.

Fuzzy rules and facts on the symptoms, different stages of HIV and other information are stored in the knowledge base. The underlying concept of the inference engine is the fuzzy logic. Thus, the inference engine performs a set of operations such as fuzzification, fuzzy inference and defuzzification, in order to produce an informative prediction. The flowchart of MAVSCOT is depicted in S2 Fig. The user interface consists of instruction versions of the software in English language and some African indigenous languages such as Afrikaans, Zulu and Xhosa, in separate sections. The voice components that pronounce HIV symptoms in English, Afrikaans, Zulu and Xhosa in separate GUI for each indigenous language, were developed in MAVSCOT.

### 2.3 Algorithm and fuzzy rule

The algorithm for MAVSCOT to diagnose follows ten steps:

Step 1: Input patient symptoms, with *s*_1_,…,*s*_*n*_ representing *n* symptoms; also include input from speech-to-text conversion.
Step 2: Weighing factors (*wf*) are assigned to describe symptoms: *wf* = 1 (mild), 2 (moderate), and 3 (severe).
Step 3: Apply fuzzy rules to the existing weighing factors of symptoms.
Step 4: Determine the Degree of Membership by comparing respective weighing factors with fuzzy inputs, by mapping. This model: $C=\{(k_{i},\mu_{C}(k_{i})|k_{i}\in V,\mu_{C}(k_{i})\in \left[0,1\right]\}$ helps to determine the degree of membership. Its description can be found in Eq 1.
Step 5: Calculate the rule base.
Step 6: Determine the execution strength of rules. This model $RSS=\sum_{x=1}^{n}R_{x}^{2}$ was useful in determining the execution strength of rules (See Eq 3 for more about the model and its description).
Step 7: Determine each rule’s degree of truth through non-zero minimum evaluation.
Step 8: Compute the possible overall severity of HIV present in a patient’s body by considering other factors in addition to the symptoms keyed-in. Defuzzification was performed using this model $\frac{\sum_{i=1}^{m}\mu K(k_{i})k_{i}}{\sum_{i=1}^{m}\mu K(k_{i})}$, in order to compute the overall HIV severity within a patient’s body and arrive at an output for the diagnosis (See Eq 4 for the model and its description).
Step 9: Output of the diagnosis with recommendation.
Step 10: Convert the results of the diagnosis from text-to-speech in English, Afrikaans, Xhosa, and Zulu.

The Internal support mechanism for the multi-lingual indigenous informatics system consists of the knowledge base, the database and the fuzzification of input variables will leverage on the merits of fuzzy logic components. The fuzzification process entails converting input value for each variable into fuzzy term from a specified set. The specified set is denoted as {mild, moderate, severe}. The contents of the specified set are defined over the variables. The class of “Mild” associated with HIV symptoms takes fuzzy values “ 0.1≤*k*<0.3 “; the class of “Moderate” with “ 0.3≤*k*<0.6 “, while the linguistic variable; and the class of “Severe” with “ 0.6≤*k*<0.8 “.

Specifically, the first step involves inputting HIV symptoms. The second step involves assigning weighing factors to each variable to depict the severity of each HIV symptom. The third step involves the establishment of fuzzy rules. MAVSCOT has a rule-based feature with different sets of IF-THEN rules. We applied the knowledge gained from the literature and interactions with medical professionals, to build the fuzzy rule base of the informatics system. The execution of a rule in the informatics system is dependent upon a symptom being classified as mild, moderate, or severe, which results in a TRUE; otherwise, no action is executed. The fourth step requires determining the degree of membership:

$$C=\{(k_{i},\mu_{C}(k_{i})|k_{i}\in V,\mu_{C}(k_{i})\in \left[0,1\right]\},$$ (1)

where *C* is the given fuzzy set, *k*_*i*_ is the HIV diagnostic variables, *μ*_*C*_ is the degree of membership of *k*_*i*_ in *C*, *V* is the set which accommodates the HIV diagnosis variables denoted by *k*_*i*_. Fuzzification process describes an event that transforms a real scalar value into its fuzzy equivalent. The process involves selecting input parameters into horizontal axis and vertical projection, in order to generate the membership degree. Fuzzification can be achieved by different types of fuzzifiers. For this research, a triangular fuzzifier was adopted:

$$\mu_{C}\left(k_{i}\right)=\{\begin{array}{ll} 1 & \mathrm{if}\;k_{i}<a\\ \frac{k_{i}-a}{b-a} & \mathrm{if}\;a\leq k_{i}<b\\ \frac{c-k_{i}}{c-b} & \mathrm{if}\;b\leq k_{i}<c\\ 0 & \mathrm{if}\;c<k_{i} \end{array}$$ (2)

where *a*, *b*, and *c* are parameters that controls the triangular shape. In practice, we have *a* = 0.1, *b* = 0.2, *c* = 0.3 for mild symptoms; *a* = 0.3, *b* = 0.45, *c* = 0.6 for moderate symptoms; *a* = 0.6, *b* = 0.7, *c* = 0.8 for severe symptoms.

The fifth step of the algorithm involves computing or calculating the rule base. The sixth step is to determine the execution strength of the rules. The logic that governs the decision-making process is controlled by an engine called the fuzzy inference engine. This operation is performed through the application of the operations, from the rule base to the values of the variables of input received. In this situation, the Root Sum Square (RSS) was applied to combine the effects of the executed rules to derive a meaningful inference. The ROOT-SUM-SQUARE (RSS) index represents an inferential method that is used in the design to help combine the effects of all applicable rules, and it also assists to perform the computation of the "fuzzy" centroid of the composite area. The RSS receives its sets of input from the Rule Base and processes them to predict the level of HIV inherent in a patient. The function of the RSS method is to integrate the effects of all rules that mattered (i.e. applicable rules). It also helps to scale the functions of the significant rules at their respective magnitude. Finally, the fuzzy centroid of the composite area is constructed. Our choice for this method was motivated by the fact that in the design, it provides the best weighted influence on all rules that are being fired or performed. The equation for RSS is as follows:

$$RSS=\sum_{x=1}^{n}R_{x}^{2}$$ (3)

where *R*_*x*_ represents a fired rule or rules that have been executed, and *x* is the identifier of rules that have been executed and represents the strength of different rules and the parameter x = 1, 2, 3,…,n also represents the number of fired rules for a given HIV diagnosis task.

The seventh step is to determine each rule’s degree of truth, through non-zero minimum evaluation. The eight step is to compute the possible level of HIV presence in a patient’s body based on the symptoms keyed-in. For the purpose of defuzzification in the informatics system, the center of gravity (CoG) or Centroid of Area (CoA) was adopted:

$$CoA=\frac{\sum_{i=1}^{m}\mu K(k_{i})k_{i}}{\sum_{i=1}^{m}\mu K(k_{i})}$$ (4)

where *μK*(*k*_*i*_) is the degree of *i* in a membership function, while *k*_*i*_ is the center value in the function. m (in Eq (4)) is the maximum value that *i* can achieve, *i* is the number of partitioned portions within the Centroid of Area (CoA). Computational flexibility and intuitive plausibility informed our choice of this methodology.

The ninth step of the algorithm is to implement and integrate text-to-speech mechanism into MAVSCOT software. We integrated the speech synthesis aspect into MAVSCOT, by incorporating some text-to-speech java classes developed by MARY TTS [56–59]. The java classes helped to translate texts within MAVSCOT into speech. MARY implies Modular Architecture for Research in sYynthesis [MARY Text-to-Speech System (MaryTTS) [59].

The tenth step of the algorithm is to output the results of the diagnosis based on the computation performed in steps 1–8 of the algorithm. It also converts the results of the diagnosis from text-to-speech in English, by applying the voice computing mechanism that was implemented in step 9 of the algorithm, to translate into Afrikaans, Xhosa, and Zulu. It then provides appropriate recommendations from text-to-speech in English, and by applying the voice computing mechanism that was implemented in step 9 of the algorithm to translate from text-to-speech, and pronounce in Afrikaans, Xhosa, and Zulu languages.

## 3 Results

### 3.1 Demonstration and examples

Some paradigms are correct about effectively managing HIV. (i) The long-term survival of an HIV patient becomes guaranteed through an early diagnosis and proper management of the disease. (ii) Proper management of HIV patients through the administration of Antiretroviral (ART/ARV) drugs and good counselling, can assist to slow down the transmission of the disease. (iii) A proper knowledge about the viral load of HIV within the human body or a similar metric, will assist clinicians and medical personnel to effectively manage such HIV patients well. In these situations, fuzzy algorithm plays a very crucial role. We demonstrate this by considering some examples. The HIV symptoms were obtained from scientific literature. This example illustrates seven patients with different HIV symptoms as illustrated in S3 Table. The patient’s IDs are: PID1, PID2, PID3, PID4, PID5, PID6, and PID7. Ultimately, our focus will be on Patient 7(PID7). Each patient has 12 HIV symptoms (see S3 Table).

To better link section 3 to section 2, we will follow the description of the algorithms in section 2. From step 1 of the MAVSCOT software algorithm in section 2, the patients HIV symptoms are input into the software. For step 2, the severity of the type of HIV symptoms for a particular HIV patient can be specified by applying weighing factors(wf) to the set S, where *wf* = 1 (mild), 2 (moderate), and 3 (severe). S3 Table shows seven HIV patients exhibiting the similar HIV symptoms but at different severity. S4 Table shows the different weights assigned to the HIV symptoms of the patients.

The third step of the MAVSCOT algorithm in section 2 focused on applying fuzzy rules to existing weighing factors of the HIV symptoms. S7 Table contains the Fuzzy rule base that has been defined. (See the online supplementary material and S7 Table). It has 21 rules, with some interpretations provided here from **Rules 1, 10, and 21** in subsequent section of this manuscript. The fourth step of the MAVSCOT algorithm involves determining the degree of membership by comparing respective weighing factors with fuzzy inputs, through mapping. For example, if an HIV patient informs a medical doctor that he or she has a severe situation of ulcer in the genitals, the medical doctor simply assigns the value 3 to this symptom. Therefore, the system will compute the degree of the ulcers in the genitals as (3–1)/3 = 2/3 = 0.67. This is achieved by using triangular fuzzifier to obtain the triangular fuzzy numbers. See S5 Table for the triangular fuzzy numbers of the HIV symptoms for the different HIV patients. Values entered for patient 7 can be found in S6 Table. The fifth step of the MAVSCOT algorithm in section 2 was to compute the rule base.

The sixth step of the MAVSCOT algorithm was to determine the execution strength of rules of patient 7 (PID7) (see S9 Table). First, the commencement of this process can be achieved from S9 Table, by extracting the set of rules that produced the non-zero minimum values. In S9 Table, we have the list of Rules that produced non-zero minimum values. These sets of rules are: Rules 1, 4, 5, 6, 7, 8, 9, 10, 11, 16, 17, 18, 19, 20, 21. These can be respectively classified as follows: Mild = None (Rules that generated non-zero minimum values); Moderate = R4, R5, R6, R8, R18 (Rules that generated non-zero minimum values); Severe = R1, R7, R9, R10, R11, R16, R17, R19, R20, R21 (Rules that generated non-zero minimum values). Second, by applying the RSS equation, the execution strength of the rules can be determined. Specifically, by comparing S8 Table with S9 Table for patient 7 (PID7), and by applying the RSS inference technique, we have:

For the class of Mild, we have that:

$$\mathrm{Mild}=0\;\sqrt{0}=\sqrt{0^{2}}=0$$ (5A)

For the class of Moderate, we have that:

$$\begin{array}{c} \mathrm{Moderate}=R9,R16,R17,R18,R20=\sqrt{R_{9}^{2}+R_{16}^{2}+R_{17}^{2}+R_{18}^{2}+R_{20}^{2}}=\\ \sqrt{0.33^{2}+0.33^{2}+0.33^{2}+0.33^{2}+0.33^{2}}=0.78 \end{array}$$ (5B)

For the class of Severe, we have that:

$$\begin{array}{l} \;Severe=R1,R4,R5,R6,R7,R8,R10,R11,R19,R21,=>\\ \;\sqrt{R_{1}^{2}+R_{4}^{2}+R_{5}^{2}+R_{6}^{2}+R_{7}^{2}+R_{8}^{2}+R_{10}^{2}+R_{11}^{2}+R_{19}^{2}+R_{21}^{2}}=\\ \;\sqrt{0.67^{2}+0.67^{2}+0.67^{2}+0.67^{2}+0.67^{2}+0.67^{2}+0.67^{2}+0.67^{2}+0.67^{2}+0.67^{2}}=\\ \sqrt{0.4489+0.4489+0.4489+0.4489+0.4489+0.4489+0.4489+0.4489+0.4489+0.4489}=2.12 \end{array}$$ (5C)

The seventh step of the MAVSCOT algorithm was to determine each rule’s degree of truth through non-zero minimum evaluation. The non-zero minimum values were computed by comparing the symptoms and values of Patient 7 with the 21-rule Fuzzy rule base. Sets of non-zero minimum values were derived (see S8 Table), from which the minimum of these non-zero minimum values were selected. In S8 and S9 Tables, a Rule-Based evaluation for Patient 7(PID7) was conducted. This was based on the Rule-base specified in S7 Table, using 21 rules. Here we have that:

Mild = Execution strength for mild for non-zero minimum values (Eq 5A) divided by the number of Rules for mild = $\frac{0}{1}$ = 0

Moderate = Execution strength for moderate for non-zero minimum values (Eq 5B) divided by the number of Rules for moderate = $\frac{0.78}{5}$ = 0.156

Severe = Execution strength for severe for non-zero minimum values (Eq 5C) divided by the number of Rules for severe = $\frac{2.12}{10}$ = 0.212;

The eighth step of the MAVSCOT algorithm involved computing the possible intensity of HIV present in a patient. The eight step helps to compute the output which is the result of the diagnoses of patient 7 (PID7). The Center of Gravity (CoG) Technique was applied and defuzzification process takes place. The CoG helped to translate the output from the RSS to crisp values. So, for patient 7(PID7), we can further apply the CoG technique to compute precise outputs for the diagnoses by adopting the defuzzification process, which gives CoG = 0.5946. Therefore, the HIV diagnoses for this male HIV patient (PID7) produced 59.46% of overall severe HIV. This was achieved as follows:

$$Output=\frac{\left(0X0.2)+(0.156X0.45)+(0.212X0.7\right)}{0+0.156+0.212}=\frac{0.22}{0.37}=0.5946$$

The output of the MAVSCOT algorithm produced 0.5946. Therefore, the overall HIV severity = 0.5946 X 100 = 59.46%. This represents an estimated overall percentage of HIV severity within a diagnosed patient’s body (see S2 Table for the interpretation of the result). The MAVSCOT software predicted **57.44%** from S7 Table, row 10, for the same type of HIV symptoms that the MAVSCOT algorithm processed. The (2.02%) slight difference obtained from the results predicted by the MAVSCOT software and the result obtained from the MAVSCOT algorithm stem from the answers provided to the additional six lifestyle follow-up questions that was integrated into the MAVSCOT software, (See S7 Table, row 10), where the lifestyle of the diagnosed patient involved having multiple sex partners, shared unsterilized objects with other persons, has had unprotected sex, uses aphrodisiac sex stimulants and knows his/her HIV status, with the assumption of being careful not to infect others. The ninth step of MAVSCOT algorithm helped to output the diagnosis with recommendation. Implementation of Step 10 of the MAVSCOT algorithm can be found in section 3.2 of this manuscript. Another example on the implementation and application of fuzzy rule for a female HIV patient can be found in S3 Appendix (with S12–S15 Tables).

**Rule 1:** IF Abnormal Swelling = Mild and Anxiety = Moderate, and Dementia = Severe, and Fatigue = Severe, and Fever = Moderate and Headache = Severe and Sexual Dysfunction = Moderate and Night Sweats = Moderate and Joint Pain = Severe and Muscle Aches = Moderate and Ulcers in the Genitals = Moderate and Weight Loss = Moderate and Patient has multiple sex partners, and Patient has shared unsterilized objects with others and Patient has had unprotected sex, and Patient has undergone unscreened blood transfusion and Patient is aware of HIV/AIDS and Patient has been self-administering sexual stimulants THEN the possible presence of HIV in the patient’s body = **SEVERE**
**Rule 10:** IF Abnormal Swelling = Severe and Anxiety = Severe, and Dementia = Severe, and Fatigue = Severe, and Fever = Severe and Headache = Severe and Sexual Dysfunction = Severe and Night Sweats = Severe and Joint Pain = Severe and Muscle Aches = Severe and Ulcers in the Genitals = Severe and Weight Loss = Severe and Patient has multiple sex partners, and Patient has shared unsterilized objects with others and Patient has had unprotected sex, and Patient has undergone unscreened blood transfusion and Patient is aware of HIV/AIDS and Patient has been self-administering sexual stimulants THEN the possible presence of HIV in the patient’s body = **SEVERE**
**Rule 21:** IF Abnormal Swelling = Mild and Anxiety = Severe, and Dementia = Severe, and Fatigue = Severe, and Fever = Moderate and Headache = Severe and Sexual Dysfunction = Moderate and Night Sweats = Moderate and Joint Pain = Severe and Muscle Aches = Moderate and Ulcers in the Genitals = Moderate and Weight Loss = Moderate and Patient has multiple sex partners, and Patient has shared unsterilized objects with others and Patient has had unprotected sex, and Patient has undergone unscreened blood transfusion and Patient is aware of HIV/AIDS and Patient has been self-administering sexual stimulants THEN the possible presence of HIV in the patient’s body **= SEVERE**

### 3.2 MAVSCOT Graphical User Interface (GUI)

The tenth step of MAVSCOT algorithm helped to convert the results of the diagnosis from text-to-speech in English, Afrikaans, Xhosa, and Zulu languages. S3 Fig shows the Graphical User Interface (GUI) of MAVSCOT, with all features displayed in English. The GUI consists of three major sections grouped into steps. These are the basic information section, the HIV symptom entry section, and other follow-up (lifestyle) questions section. An extra supplementary section represents the voice detection programmed into the MAVSCOT GUI. MAVSCOT’s GUIs in Afrikaans, Zulu and Xhosa can be found in S4–S6 Figs, respectively. Other figures can be found in the S2 Appendix.

### 3.3 Gaps in the existing systems

Comparative analysis between MAVSCOT and some existing systems revealed the gaps inherent in the existing systems. Some of the gaps inherent in the existing systems include the followings: (i) the existing systems developed by Pazzani et al. [33], Tucker et al. [37], Ebrahimi et al. [36], Atalay et al. [34], all lacked multilingual features of languages used in developing the systems. (ii) another gaps inherent in the existing system is that the systems developed by Ebrahimi et al. [36], Pazzani et al. [33], and Atalay et al. [34] lacked voice-enabled/speech enabled features in their operations.(iii) the systems developed by Tucker et al. [37], Ebrahimi et al. [36] and Atalay et al. [34] lacked HIV predictive and advisory features. See S11 Table.

### 3.4 Gaps inherent in MAVSCOT

MAVSCOT has its limitations. The speech pronunciation of HIV symptoms in English, Afrikaans, Zulu and IsiXhosa, within the symptoms section, the diagnosed results section, and the prescription section of the MAVSCOT Software yielded very good and impressive results. However, the ascent and pronunciation of the spoken sentences in the advice and recommendation sections of the Afrikaans, Zulu and IsiXhosa versions of MAVSCOT still have some defects. There is the need to improve these ascents and pronunciation in future works.

## 4 Discussion

We conducted some comparisons between existing systems and the MAVSCOT software. S11 Table shows the similarities and differences between the listed systems. When compared with other voice-related software, MAVSCOT is the only software that has multilingual features in three indigenous languages (Afrikaans, IsiXhosa and Zulu). Although MAVSCOT is speech-based and voice-enabled software, it is not an interactive speech-based system. MAVSCOT and the system developed by Pazzani et al. [33], have HIV predictive and advisory features. Indigenous health informatics toolkits are very rare in Africa, especially in the management of incurable diseases such as HIV. Indigenous health informatics toolkits with embedded enabled speech features, will complement the efforts of health practitioners and people in sub-urban, and rural communities of Africa, in the management of HIV.

Fuzzy logic has a way of representing, reprocessing, manipulating, and handling data that are characterized with uncertainty and vagueness, and then producing intelligent reasoning out of it, for the purpose of making informed decision [60–62]. Diseases have different progressive stages [63]. During disease diagnosis, based on available symptoms, intelligent decisions are required, to decipher the correct stage of such disease. This could provide platforms for prescribing the correct medication or proffer the correct measures to manage such diseases. Fuzzy logic has the capacity to decipher specific stages of diseases. When fuzzy logic is combined with or integrated into language technology, the positive impact can be tremendous. Integrating the predictive capability of Fuzzy logic in disease diagnosis, coupled with language technology, can go a long way to transcending barriers and limitations [64–66]. This integration can assist in managing diseases like HIV and help drastically reduce the transmissions.

Overall, we hope MAVSCOT can help improve diagnosing HIV patients in rural communities of Africa, which could eventually avert and reduce the transmissions of new HIV infections. This is the first indigenous HIV multilingual software to integrate the diagnostic and predictive knowledge of fuzzy logic [65], fuzzy sets [66], fuzzification and defuzzification [67–70], with selected indigenous African languages, as well embedded text-to-speech function. It can be used as a valuable complementary tool to support medical personnel in indigenous communities in Africa. MAVSCOT can be further expanded to include other indigenous languages such as SiSwati, Xitsonga, Setswana and Tshivenḓa. The speech detection component in MAVSCOT also needs further refinement. However, our priority for the future is to test the system by using raw HIV Clinical health data records.

## 5 Conclusion

MAVSCOT was developed by applying the knowledge of fuzzy sets, fuzzy logic and speech technology, to develop a multi-lingual indigenous informatics system (MAVSCOT), in four different languages namely: English, Afrikaans, Zulu and IsiXhosa. MAVSCOT is a complementary tool that will assist medical personnel to facilitate effective HIV diagnosis and prediction, in underprivileged rural and sub-urban communities of Africa. The positive impact of the use of this software by medical practitioners in rural settings of Africa will greatly assist to reduce time wasted on HIV diagnosis among local patients, eliminate miscommunication, ensure better diagnosis, treatments, and management of HIV. This will assist to reduce the rate of transmission of the disease. Hence, HIV/AIDs transmissions will be delayed, thus prolonging the lives of HIV infected people, and protecting the lives of the uninfected.

## Supporting information

S1 Fig Schematic depiction of the architecture of the Multi-lingual Indigenous HIV Informatics System (MAVSCOT). This architecture shows the interaction between the knowledge base, working memory and the inference engine. The architecture also shows how knowledge engineering was used to transform medical expertise and knowledge gathered, into coding the MAVSCOT expert system. The users of MAVSCOT interacts with the system through voice-enabled GUI.
(TIF)

S2 Fig Flowchart of the Multi-lingual Indigenous HIV Informatics System (MAVSCOT). This flowchart depicts the various operations within MAVSCOT.
(TIF)

S3 Fig English Graphical User Interface (GUI) of the MAVSCOT software. This is the English user interface of MAVSCOT provides a description of the different sections/segments within the MAVSCOT software GUI.
(TIF)

S4 Fig The Afrikaans language Graphical User Interface of the multi-lingual HIV indigenous fuzzy logic based diagnostic system (MAVSCOT). This GUI provides the description of the various segments/sections of the interface in the Afrikaans language. The Afrikaans GUI Is also a multilingual HIV voice-enabled software (specifically in Afrikaans language).
(TIF)

S5 Fig The IsiXhosa language Graphical User Interface of the multi-lingual HIV indigenous fuzzy logic based diagnostic system (MAVSCOT). This GUI provides the description of the various segments/sections of the interface in the IsiXhosa language. The IsiXhosa GUI Is also a multilingual HIV voice-enabled software (specifically in IsiXhosa language).
(TIF)

S6 Fig The Zulu language Graphical User Interface of the multi-lingual HIV indigenous fuzzy logic based diagnostic system (MAVSCOT). This GUI provides the description of the various segments/sections of the interface in the Zulu language. The Zulu GUI is also a multilingual HIV voice-enabled software (specifically in Zulu language).
(TIF)

S1 Table MAVSCOT HIV symptoms of HIV patients obtained from medical and scientific literature. This table provides description about HIV patient symptomatic data, as obtained from different and exiting literature, the in-text citations for the literature. The columns consist of the collection samples, HIV Symptoms of patients [PLWHIV] obtained from medical and scientific literature and the references for each collection sample.
(DOC)

S2 Table Different stages of HIV, possible interpretation of HIV predicted values and range. This table consists of different Stages of HIV and the corresponding literature to support these stages.
(DOC)

S3 Table Sample data from patients with 12 HIV symptoms. This table consists of HIV symptoms for seven different HIV patients. The table consists of information about the patients’ IDs, gender and HIV symptoms.
(DOC)

S4 Table Weights assigned to HIV symptoms of HIV patients by doctors who have interacted with the patients concerned. This table shows a sample of rating of the patients on HIV diagnosis variables. This table shows the weights assigned to patients by doctors who have interacted with the patients concerned.
(DOC)

S5 Table Derived triangular values for HIV symptoms for patient 7, using MAVSCOT. This table shows the derived triangular values for the HIV symptoms for patient 7, within MAVSCOT software.
(DOC)

S6 Table Values entered for patient 7 (with ID = PID7). This table consists of HIV symptoms, degree of HIV symptoms, and the values of Triangular fuzzy numbers of the HIV symptoms.
(DOC)

S7 Table Fuzzy rule base for HIV–using 21 rules. This table consists of rule numbers, different severities of HIV symptoms and the predicted diagnosis for HIV patients at different HIV symptom severities; and the conclusion of the overall HIV diagnosed.
(DOC)

S8 Table Rule-based evaluation for patient 7 (PID7). This table shows a Rule-Based evaluation for Patient 7 (PID7), based on the Rule base specified–using 21 rules. The table shows the final outcome of the Non-zero minimum values.
(DOC)

S9 Table List of rules that produced non-zero minimum values. This table shows the sets of rules that met the Non-zero minimum values criteria. These are: Rules 1, 4, 5, 6, 7, 8, 9, 10, 11, 16, 17, 18, 19, 20, 21. These can be respectively classified as follows: Mild = None; Moderate = R4, R5, R6, R8, R18, R19; Severe = R1, R7, R9, R10, R11, R16, R17, R19, R20, R21.
(DOC)

S10 Table Predicted HIV diagnosis results by MAVSCOT for the patient. This table shows the results produced by MAVSCOT in English, Afrikaans, IsiXhosa and Zulu languages. These are the percentage of overall HIV severity diagnosed per patient.
(DOC)

S11 Table Comparison of existing HIV voice-enabled expert system/software with HIV multilingual indigenous informatics software. This table provides a comparative analysis between MAVSCOT software and other existing HIV voice-enabled expert system/software. The metrics used for this comparison include the description, multi-lingual features (text-based), Voice-Enabled/Speech-based features, Functionalities—HIV Predictive Feature, Advisory Features, input and output data.
(DOC)

S12 Table Fuzzy rule base for the HIV multilingual informatics software in English and three (3) indigenous South African languages–using 14 rules. This table is the Fuzzy Rule-Base table for a second HIV patient. This table consists of an expanded number of HIV symptoms (24 HIV symptoms), with 14 rules. The table also provided answers to some of the lifestyle questions in MAVSCOT, before determining the diagnosis of the severity of the HIV of the second patient. Most of the results in this table, show that the HIV diagnosis is SEVERE unlike the table for the first HIV patient that had most of the diagnosed cases of HIV diagnosis as MODERATE.
(DOC)

S13 Table Example of symptoms, severity, rating on variables of 24 HIV symptoms of another patient. This table provides information about the 24 HIV symptoms of a second patient used as illustration for the demonstration of the diagnosis of the MAVSCOT software. This table consists of the rating on the variables, and the triangular fuzzification function values.
(DOC)

S14 Table Triangular fuzzy function values. This table shows the Triangular Fuzzy Function Values.
(DOC)

S15 Table Table of 24 HIV symptoms, non-zero minimum values and HIV diagnosis. This table shows the 24 HIV symptoms, and how the Fuzzy Rule-base values have been able to produce HIV diagnosis and their non-zero minimum values.
(DOC)

S1 Appendix Multilingual HIV symptoms, words, sentences, labels used in MAVSCOT software. This file consist of all HIV symptoms used in MAVSCOT, the labels, words, sentences used in constructing and developing MAVSCOT. This file also contains the interpretations for the HIV symptoms, words, sentences in Afrikaans, IsiXhosa and the Zulu indigenous African languages.
(XLSX)

S2 Appendix All figures used in the manuscript and the supporting documents. This file contains all figures used in the main manuscript and supporting documents.
(DOC)

S3 Appendix Extra example to illustrate the implementation of fuzzy logic and fuzzy rule within MAVSCOT. This file contains an extra example to illustrate the implementation of fuzzy rule within the MAVSCOT software.
(DOC)

S1 File All tables, figures, and illustrations within MAVSCOT. This file consists of all tables, figures, description, and illustration of an extra example on the use of MAVSCOT software.
(DOC)

S2 File (EXE)

S1 Data (ZIP)

S1 Video (MP4)

S2 Video (MP4)

S3 Video (MP4)

S4 Video (MP4)
