# Supplementary figures and images for: MAVSCOT: A fuzzy logic-based HIV diagnostic system with indigenous multi-lingual interfaces for rural Africa
Source: PLoS One. 2020 Nov 6;15(11):e0241864. doi: 10.1371/journal.pone.0241864 (PMC7647102; doi:10.1371/journal.pone.0241864)

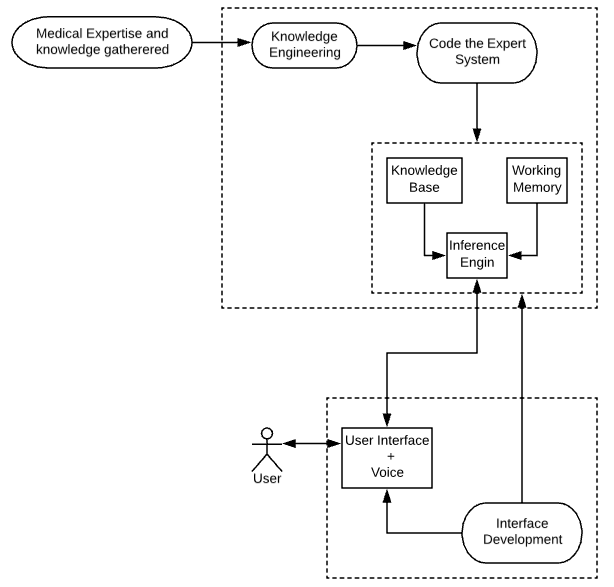

Supplement: S1 Fig — This architecture shows the interaction between the knowledge base, working memory and the inference engine. The architecture also shows how knowledge engineering was used to transform medical expertise and knowledge gathered, into coding the MAVSCOT expert system. The users of MAVSCOT interacts with the system through voice-enabled GUI. (TIF) [file pone.0241864.s001.tif]

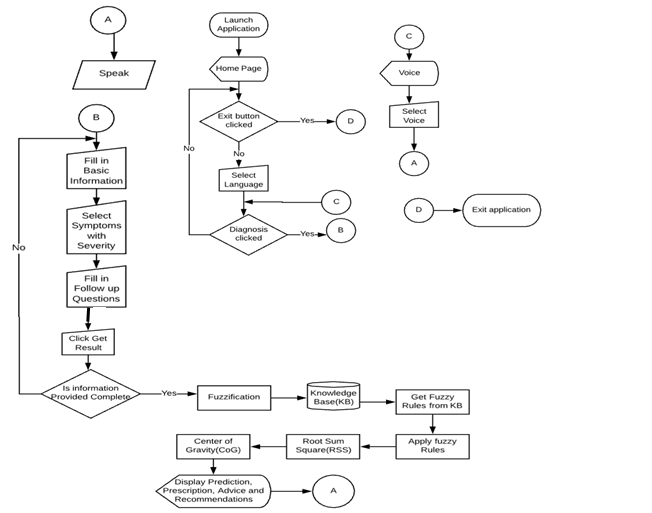

Supplement: S2 Fig — This flowchart depicts the various operations within MAVSCOT. (TIF) [file pone.0241864.s002.tif]

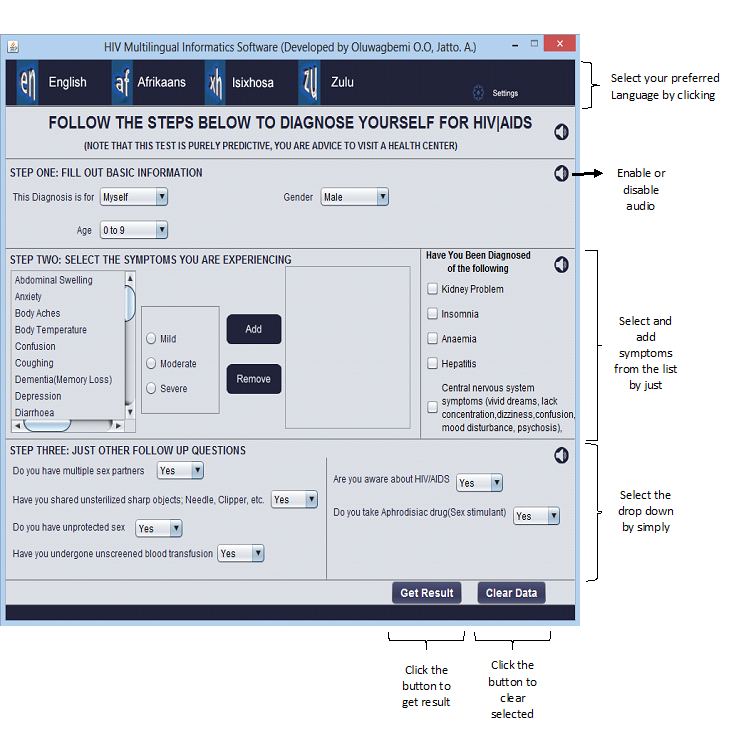

Supplement: S3 Fig — This is the English user interface of MAVSCOT provides a description of the different sections/segments within the MAVSCOT software GUI. (TIF) [file pone.0241864.s003.tif]

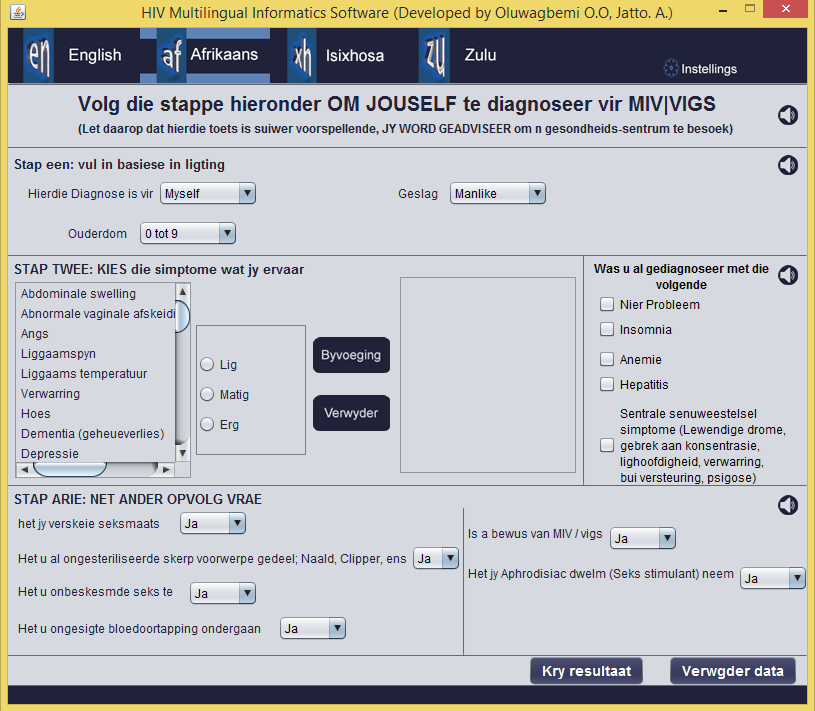

Supplement: S4 Fig — This GUI provides the description of the various segments/sections of the interface in the Afrikaans language. The Afrikaans GUI Is also a multilingual HIV voice-enabled software (specifically in Afrikaans language). (TIF) [file pone.0241864.s004.tif]

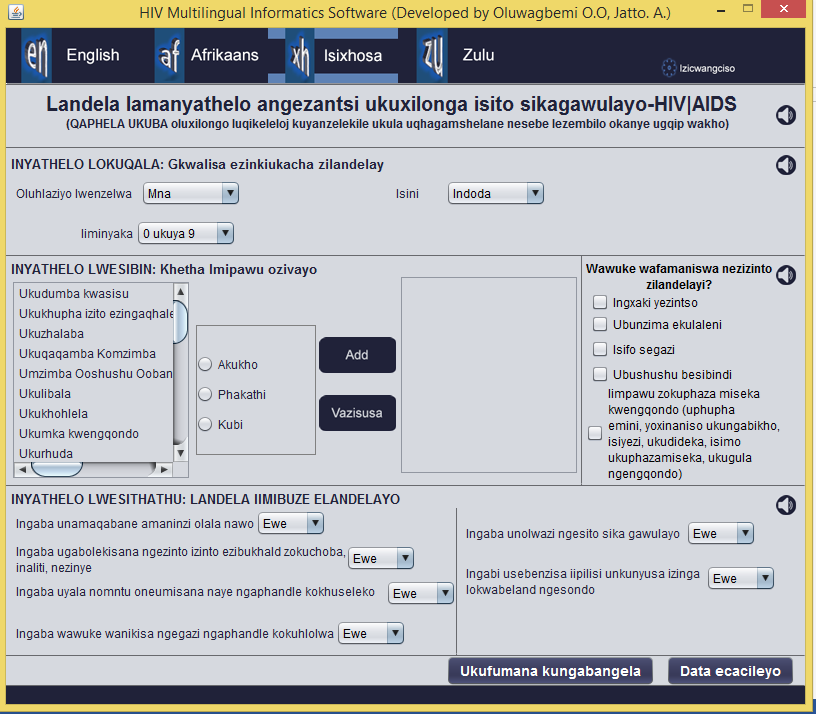

Supplement: S5 Fig — This GUI provides the description of the various segments/sections of the interface in the IsiXhosa language. The IsiXhosa GUI Is also a multilingual HIV voice-enabled software (specifically in IsiXhosa language). (TIF) [file pone.0241864.s005.tif]

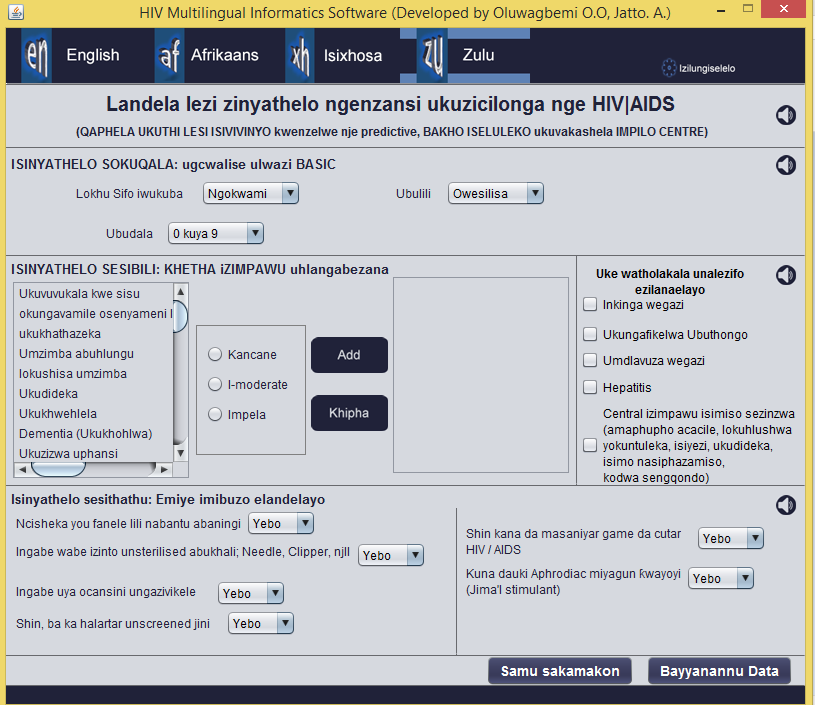

Supplement: S6 Fig — This GUI provides the description of the various segments/sections of the interface in the Zulu language. The Zulu GUI is also a multilingual HIV voice-enabled software (specifically in Zulu language). (TIF) [file pone.0241864.s006.tif]
