# Supplementary material for: MAVSCOT: A fuzzy logic-based HIV diagnostic system with indigenous multi-lingual interfaces for rural Africa
Source: PLoS One. 2020 Nov 6;15(11):e0241864. doi: 10.1371/journal.pone.0241864 (PMC7647102; doi:10.1371/journal.pone.0241864)
Supplement: S1 Table — This table provides description about HIV patient symptomatic data, as obtained from different and exiting literature, the in-text citations for the literature. The columns consist of the collection samples, HIV Symptoms of patients [PLWHIV] obtained from medical and scientific literature and the references for each collection sample. (DOC) [file pone.0241864.s007.doc]

**S1 Table: MAVSCOT HIV Symptoms of HIV patients obtained from medical and scientific literature**

| **Collection of HIV symptomatic Data** | **HIV Symptoms of patients [PLWHIV] obtained from medical and scientific literature** | **References** |
| --- | --- | --- |
| **Collection 1** | Poor sleep, Muscle aches/Joint pain, Fatigue, Anxiety/Nervous, Sadness, Numbness/Pain in the feet, Headache, Memory loss, Sex Problems, Cough,/Shortness of breath, Fever/Chills, Sweats, Dizzy/Dizziness, Bloating/Abdominal pain, Poor appetite, Diarrhea, Nausea/Vomiting, Loss of appetite, Rash, Weight Loss | Wilson et. al., 2016 |
| **Collection 2** | Fatigue, Fevers, Dizziness,Hand/foot pain, Memory loss, Nausea, Diarrhea, Sadness, Sleep trouble, Skin problems Cough, Headache, Sweats/chills, Weight loss, Appetite loss, Eye trouble, Mouth pain, Mouth infection, Chest pain, Trouble breathing Runny nose, Abdominal pain, Trouble urinating, Muscle/joint pain | Justice et al., 2001 |
| **Collection 3** | Symptoms frequently reported by People Living with HIV(PLWHIV):  Abdominal pain, Anxiety, Changes in body weight/fat, Cognitive decline, Diarrhea, Fatigue, Fever or night sweats, Headaches, Insomnia, Joint pain/stiffness, Loss of strength, Muscle pain, Nausea/vomiting ,Peripheral neuropathy, Reduction in appetite, Sadness, Sexual problems, Shortness of breath/cough, Skin problems | Wilson et. al., 2014 |
| **Collection 4** | Trouble falling asleep, Weakness, Numbness/tingling, Poor appetite, Nausea/upset stomach, Hot or cold spells, Shortness of breath,  Faintness/dizziness, Pain in heart/chest | Hudson et al., 2003 |
| **Collection 5** | Dementia | Ghafouri et al., 2006 |

This table provides description about HIV patient symptomatic data, as obtained from different and exiting literature, the in-text citations for the literature. The columns consist of the collection samples, HIV Symptoms of patients [PLWHIV] obtained from medical and scientific literature and the references for each collection sample.
