# Supplementary material for: MAVSCOT: A fuzzy logic-based HIV diagnostic system with indigenous multi-lingual interfaces for rural Africa
Source: PLoS One. 2020 Nov 6;15(11):e0241864. doi: 10.1371/journal.pone.0241864 (PMC7647102; doi:10.1371/journal.pone.0241864)
Supplement: S2 Table — This table consists of different Stages of HIV and the corresponding literature to support these stages. (DOC) [file pone.0241864.s008.doc]

**S2 Table. Different Stages of HIV, possible interpretation of HIV predicted values and range**

| **Software Predicted Percentage Range** | **Software Degree of HIV intensity** | **Possible HIV Stage in the Medical field** |
| --- | --- | --- |
| 0%-24% | Mild | Acute HIV Infection –sharp drop in concentration of circulating CD4+T cells (Hernandez-Vargas and Middleton, 2013) |
| 25%-49% | Moderate | Chronic HIV Infection(Clinical latency) CD4+T cells circulation – near normal, drop in viral loads (Hernandez-Vargas and Middleton, 2013) |
| 50%-74% | Severe | Chronic HIV infection(Clinical latency) CD4+T cells circulation – near normal, drop in viral loads (Hernandez-Vargas and Middleton, 2013) |
| 75%-100% | Very Severe | Full Blown AIDS (drop in viral loads)/Full-blown AIDS(most severe stage) – CD4 < 200 Cells/mm3 (Hernandez-Vargas and Middleton, 2013) |

This table consists of different Stages of HIV and the corresponding literature to support these stages.
