# Supplementary material for: MAVSCOT: A fuzzy logic-based HIV diagnostic system with indigenous multi-lingual interfaces for rural Africa
Source: PLoS One. 2020 Nov 6;15(11):e0241864. doi: 10.1371/journal.pone.0241864 (PMC7647102; doi:10.1371/journal.pone.0241864)
Supplement: S3 Table — This table consists of HIV symptoms for seven different HIV patients. The table consists of information about the patients’ IDs, gender and HIV symptoms. (DOC) [file pone.0241864.s009.doc]

**S3 Table. Sample data from Patients with 12 HIV symptoms**

| **S/N** | **Patient’s ID** | **Gender** | **HIV Symptoms** |
| --- | --- | --- | --- |
| 1 | PID1 | Male | Abnormal swelling  Anxiety  Dementia  Fatigue  Fever  Headache  Sexual dysfunction  Night sweats  Joint Pain (Rheumatism  Muscle aches  Ulcers in the Genitals  Weight loss  12 Symptoms |
| 2 | PID2 | Female |
| 3 | PID3 | Male |
| 4 | PID4 | Female |
| 5 | PID5 | Female |
| 6 | PID6 | Male |
| **7** | **PID7** | **Male** |

This table consists of HIV symptoms for seven different HIV patients. The table consists of information about the patients’ IDs, gender and HIV symptoms.
