# Supplementary material for: MAVSCOT: A fuzzy logic-based HIV diagnostic system with indigenous multi-lingual interfaces for rural Africa
Source: PLoS One. 2020 Nov 6;15(11):e0241864. doi: 10.1371/journal.pone.0241864 (PMC7647102; doi:10.1371/journal.pone.0241864)
Supplement: S4 Table — This table shows a sample of rating of the patients on HIV diagnosis variables. This table shows the weights assigned to patients by doctors who have interacted with the patients concerned. (DOC) [file pone.0241864.s010.doc]

**S4 Table. Weights assigned to HIV symptoms of HIV patients by doctors who have interacted with the patients concerned.**

| Patient ID | Abnormal swelling | Anxiety | Dementia | Fatigue | Fever | Headache | Sexual dysfunction | Night sweats | Joint Pain (Rheumatism | Muscle aches | Ulcers in the Genitals | Weight loss |
| --- | --- | --- | --- | --- | --- | --- | --- | --- | --- | --- | --- | --- |
| PID1 | 3 | 3 | 2 | 3 | 1 | 3 | 3 | 1 | 1 | 3 | 1 | 3 |
| PID2 | 2 | 3 | 1 | 2 | 3 | 1 | 2 | 3 | 1 | 2 | 3 | 1 |
| PID3 | 3 | 1 | 1 | 1 | 1 | 1 | 1 | 1 | 1 | 1 | 1 | 1 |
| PID4 | 3 | 2 | 3 | 2 | 3 | 2 | 3 | 2 | 3 | 2 | 1 | 3 |
| PID5 | 3 | 2 | 1 | 3 | 2 | 1 | 3 | 2 | 1 | 3 | 2 | 1 |
| PID6 | 2 | 1 | 2 | 1 | 2 | 1 | 2 | 1 | 2 | 1 | 2 | 1 |
| **PID7** | **3** | **3** | **2** | **3** | **3** | **3** | **3** | **3** | **3** | **3** | **3** | **1** |

This table shows a sample of rating of the patients on HIV diagnosis variables. This table shows the weights assigned to patients by doctors who have interacted with the patients concerned.
