# Supplementary material for: MAVSCOT: A fuzzy logic-based HIV diagnostic system with indigenous multi-lingual interfaces for rural Africa
Source: PLoS One. 2020 Nov 6;15(11):e0241864. doi: 10.1371/journal.pone.0241864 (PMC7647102; doi:10.1371/journal.pone.0241864)
Supplement: S5 Table — This table shows the derived triangular values for the HIV symptoms for patient 7, within MAVSCOT software. (DOC) [file pone.0241864.s011.doc]

**S5 Table. Derived triangular values for HIV symptoms for patient 7, using MAVSCOT**

| Patient ID | Abnormal swelling | Anxiety | Dementia | Fatigue | Fever | Headache | Sexual dysfunction | Night sweats | Joint Pain (Rheumatism | Muscle aches | Ulcers in the Genitals | Weight loss |
| --- | --- | --- | --- | --- | --- | --- | --- | --- | --- | --- | --- | --- |
| PID1 | 0.67 | 0.67 | 0.33 | 0.67 | 0 | 0.67 | 0.67 | 0 | 0 | 0.67 | 0 | 0.67 |
| PID2 | 0.33 | 0.67 | 0 | 0.33 | 0.67 | 0 | 0.33 | 0.67 | 0 | 0.33 | 0.67 | 0 |
| PID3 | 0.67 | 0 | 0 | 0 | 0 | 0 | 0 | 0 | 0 | 0 | 0 | 0 |
| PID4 | 0.67 | 0.33 | 0.67 | 0.33 | 0.67 | 0.33 | 0.67 | 0.33 | 0.67 | 0.33 | 0 | 0.67 |
| PID5 | 0.67 | 0.33 | 0 | 0.67 | 0.33 | 0 | 0.67 | 0.33 | 0 | 0.67 | 0.33 | 0 |
| PID6 | 0.33 | 0 | 0.33 | 0 | 0.33 | 0 | 0.33 | 0 | 0.33 | 0 | 0.33 | 0 |
| **PID7** | **0.67** | **0.67** | **0.33** | **0.67** | **0.67** | **0.67** | **0.67** | **0.67** | **0.67** | **0.67** | **0.67** | **0** |

This table shows the derived triangular values for the HIV symptoms for patient 7, within MAVSCOT software.
