# Supplementary material for: MAVSCOT: A fuzzy logic-based HIV diagnostic system with indigenous multi-lingual interfaces for rural Africa
Source: PLoS One. 2020 Nov 6;15(11):e0241864. doi: 10.1371/journal.pone.0241864 (PMC7647102; doi:10.1371/journal.pone.0241864)
Supplement: S6 Table — This table consists of HIV symptoms, degree of HIV symptoms, and the values of Triangular fuzzy numbers of the HIV symptoms. (DOC) [file pone.0241864.s012.doc]

**S6 Table. Values entered for patient 7 (with ID = PID7).**

| **HIV Symptoms for Patient (PID7)** | **Degree of HIV symptom** | Triangular fuzzy numbers of the HIV symptoms |
| --- | --- | --- |
| Abnormal swelling | Severe | 0.67 |
| Anxiety | Severe | 0.67 |
| Dementia | Moderate | 0.33 |
| Fatigue | Severe | 0.67 |
| Fever | Severe | 0.67 |
| Headache | Severe | 0.67 |
| Sexual dysfunction | Severe | 0.67 |
| Night sweats | Severe | 0.67 |
| Joint Pain (Rheumatism) | Severe | 0.67 |
| Muscle aches | Severe | 0.67 |
| Ulcers in the Genitals | Severe | 0.67 |
| Weight loss | Mild | 0 |

This table consists of HIV symptoms, degree of HIV symptoms, and the values of Triangular fuzzy numbers of the HIV symptoms.
