# Supplementary material for: MAVSCOT: A fuzzy logic-based HIV diagnostic system with indigenous multi-lingual interfaces for rural Africa
Source: PLoS One. 2020 Nov 6;15(11):e0241864. doi: 10.1371/journal.pone.0241864 (PMC7647102; doi:10.1371/journal.pone.0241864)
Supplement: S7 Table — This table consists of rule numbers, different severities of HIV symptoms and the predicted diagnosis for HIV patients at different HIV symptom severities; and the conclusion of the overall HIV diagnosed. (DOC) [file pone.0241864.s013.doc]

**S7 Table. Fuzzy Rule Base for HIV – using 21 rules**

| Rule No. | Abnormal swelling | Anxiety | Dementia | Fatigue | Fever | Headache | Sexual dysfunction | Night sweats | Joint Pain (Rheumatism | Muscle aches | Ulcers in the Genitals | Weight loss | ALL Life Style Questions | Conclusion  HIV presence predicted |
| --- | --- | --- | --- | --- | --- | --- | --- | --- | --- | --- | --- | --- | --- | --- |
| 1 | Mild | Moderate | Severe | Severe | Moderate | Severe | Moderate | Moderate | Severe | Moderate | Moderate | Moderate | Yes | 55.32% HIV Severe |
| 2 | Moderate | Moderate | Mild | Mild | Mild | Mild | Mild | Mild | Mild | Mild | Mild | Mild | Yes | 44.72% HIV Moderate |
| 3 | Mild | Mild | Mild | Mild | Mild | Mild | Mild | Mild | Mild | Mild | Mild | Mild | Yes | 12.44 HIV Mild |
| 4 | Moderate | Mild | Mild | Mild | Mild | Mild | Severe | Severe | Severe | Severe | Severe | Severe | Yes | 46.55% HIV Moderate |
| 5 | Mild | Severe | Mild | Severe | Mild | Severe | Mild | Severe | Mild | Mild | Mild | Mild | Yes | 44.27% HIV Moderate |
| 6 | Mild | Severe | Mild | Severe | Mild | Severe | Severe | Mild | Severe | Mild | Severe | Mild | Yes | 48.57% HIV Moderate |
| 7 | Severe | Mild | Severe | Mild | Severe | Mild | Severe | Mild | Severe | Severe | Severe | Severe | Yes | 50.21  % HIV Severe |
| 8 | Mild | Mild | Mild | Mild | Severe | Mild | Severe | Mild | Severe | Mild | Mild | Mild | Yes | 47.29% HIV Moderate |
| 9 | Moderate | Moderate | Moderate | Moderate | Moderate | Moderate | Moderate | Moderate | Moderate | Moderate | Moderate | Moderate | Yes | 51.54% HIV Severe |
| 10 | Severe | Severe | Moderate | Severe | Severe | Severe | Severe | Severe | Severe | Severe | Severe | Mild | Yes | 57.44% HIV Severe |
| 11 | Mild | Mild | Severe | Severe | Mild | Mild | Severe | Severe | Mild | Mild | Severe | Mild | Yes | 50.51% HIV Severe |
| 12 | Mild | Moderate | Mild | Moderate | Mild | Moderate | Mild | Moderate | Mild | Moderate | Mild | Moderate | Yes | 44.84% HIV Moderate |
| 13 | Mild | Mild | Mild | Mild | Mild | Moderate | Mild | Mild | Moderate | Mild | Mild | Mild | Yes | 42.05% HIV Moderate |
| 14 | Mild | Mild | Mild | Mild | Mild | Mild | Mild | Mild | Mild | Mild | **Moderate** | Mild | Yes | 35.58% HIV Moderate |
| 15 | Mild | Moderate | Severe | Mild | Moderate | Severe | Mild | Moderate | Severe | Mild | Moderate | Severe | Yes | 47.5% HIV Moderate |
| 16 | Severe | Mild | Moderate | Severe | Mild | Moderate | Severe | Mild | Moderate | Severe | Mild | Moderate | Yes | 52.57% HIV Severe |
| 17 | Moderate | Moderate | Moderate | Mild | Mild | Mild | Severe | Severe | Severe | Moderate | Moderate | Moderate | Yes | 54.19% HIV Severe |
| 18 | Mild | Severe | Moderate | Mild | Severe | Moderate | Mild | Severe | Moderate | Mild | Severe | Moderate | Yes | 47.28% HIV Moderate |
| 19 | Moderate | Mild | Mild | Severe | Mild | Mild | Moderate | Mild | Mild | Severe | Mild | Mild | Yes | 47.81% HIV Moderate |
| 20 | Severe | Moderate | Moderate | Severe | Moderate | Moderate | Severe | Moderate | Moderate | Severe | Moderate | Moderate | Yes | 55.75% HIV Severe |
| 21 | Mild | Severe | Severe | Moderate | Severe | Severe | Mild | Severe | Severe | Moderate | Severe | Severe | Yes | 52.72% HIV Severe |

This table consists of rule numbers, different severities of HIV symptoms and the predicted diagnosis for HIV patients at different HIV symptom severities. ; and the conclusion of the overall HIV diagnosed.

From Table S7, some interpretations were provided for some of the rules (Rules, 1, 10, and 21) as follows:

**Rule 1:** IF Abnormal Swelling = Mild and Anxiety = Moderate, and Dementia = Severe, and Fatigue = Severe, and Fever = Moderate and Headache = Severe and Sexual Dysfunction = Moderate and Night Sweats = Moderate and Joint Pain = Severe and Muscle Aches = Moderate and Ulcers in the Genitals = Moderate and Weight Loss = Moderate and Patient has multiple sex partners, and Patient has shared unsterilized objects with others and Patient has had unprotected sex, and Patient has undergone unscreened blood transfusion and Patient is aware of HIV/AIDS and Patient has been self-administering sexual stimulants THEN the possible presence of HIV in the patients body = **SEVERE**

**Rule 10:** IF Abnormal Swelling = Severe and Anxiety = Severe, and Dementia = Severe, and Fatigue = Severe, and Fever = Severe and Headache = Severe and Sexual Dysfunction = Severe and Night Sweats = Severe and Joint Pain = Severe and Muscle Aches = Severe and Ulcers in the Genitals = Severe and Weight Loss = Severe and Patient has multiple sex partners, and Patient has shared unsterilized objects with others and Patient has had unprotected sex, and Patient has undergone unscreened blood transfusion and Patient is aware of HIV/AIDS and Patient has been self-administering sexual stimulants THEN the possible presence of HIV in the patients body = **SEVERE**

**Rule 21:** IF Abnormal Swelling = Mild and Anxiety = Severe, and Dementia = Severe, and Fatigue = Severe, and Fever = Moderate and Headache = Severe and Sexual Dysfunction = Moderate and Night Sweats = Moderate and Joint Pain = Severe and Muscle Aches = Moderate and Ulcers in the Genitals = Moderate and Weight Loss = Moderate and Patient has multiple sex partners, and Patient has shared unsterilized objects with others and Patient has had unprotected sex, and Patient has undergone unscreened blood transfusion and Patient is aware of HIV/AIDS and Patient has been self-administering sexual stimulants THEN the possible presence of HIV in the patients body = **SEVERE**
