# Supplementary material for: MAVSCOT: A fuzzy logic-based HIV diagnostic system with indigenous multi-lingual interfaces for rural Africa
Source: PLoS One. 2020 Nov 6;15(11):e0241864. doi: 10.1371/journal.pone.0241864 (PMC7647102; doi:10.1371/journal.pone.0241864)
Supplement: S8 Table — This table shows a Rule-Based evaluation for Patient 7 (PID7), based on the Rule base specified–using 21 rules. The table shows the final outcome of the Non-zero minimum values. (DOC) [file pone.0241864.s014.doc]

S8 Table. Rule-Based evaluation for Patient 7(PID7), based on the Rule base specified– using 21 rules

| Rule No. | Abnormal swelling | Anxiety | Dementia | Fatigue | Fever | Headache | Sexual dysfunction | Night sweats | Joint Pain (Rheumatism | Muscle aches | Ulcers in the Genitals | Weight loss | Non-Zero Mimimum Values |
| --- | --- | --- | --- | --- | --- | --- | --- | --- | --- | --- | --- | --- | --- |
| 1 | - | - | - | 0.67 | - | 0.67 | - | - | 0.67 | - | - | - | 0.67 |
| 2 | - | - | - | - | - | - | - | - | - | - | - | 0 |  |
| 3 | - | - | - | - | - | - | - | - | - | - | - | 0 |  |
| 4 | - | - | - | - | - | - | 0.67 | 0.67 | 0.67 | 0.67 | 0.67 | - | 0.67 |
| 5 | - | 0.67 | - | 0.67 | - | 0.67 | - | 0.67 | - | - | - | 0 | 0.67 |
| 6 | - | 0.67 | - | 0.67 | - | 0.67 | 0.67 | - | 0.67 | - | 0.67 | 0 | 0.67 |
| 7 | 0.67 | - | - | - | 0.67 | - | 0.67 | - | 0.67 | 0.67 | 0.67 | - | 0.67 |
| 8 | - | - | - | - | 0.67 | - | 0.67 | - | 0.67 | - | - | 0 | 0.67 |
| 9 | - | - | 0.33 | - | - | - | - | - | - | - | - | - | 0.33 |
| 10 | 0.67 | 0.67 | - | 0.67 | 0.67 | 0.67 | 0.67 | 0.67 | 0.67 | 0.67 | 0.67 | - | 0.67 |
| 11 | - | - | 0.67 | 0.67 | - | - | 0.67 | 0.67 | - | - | 0.67 | 0 |  |
| 12 | - | - | - | - | - | - | - | - | - | - | - | - |  |
| 13 | - | - | - | - | - | - | - | - | - | - | - | 0 |  |
| 14 | - | - | - | - | - | - | - | - | - | - | - | 0 |  |
| 15 | - | - | - | - | - | - | - | - | - | - | - | - |  |
| 16 | 0.67 | - | 0.33 | 0.67 | - | - | 0.67 | - | - | 0.67 | - | - | 0.33 |
| 17 | - | - | 0.33 | - | - | - | 0.67 | 0.67 | 0.67 | - | - | - | 0.33 |
| 18 | - | 0.67 | 0.33 | - | 0.67 | - | - | 0.67 | - | - | - | - | 0.33 |
| 19 | - | - | - | 0.67 | - | - | - | - | - | 0.67 | - | 0 | 0.67 |
| 20 | 0.67 | - | 0.33 | 0.67 | - | - | 0.67 | - | - | 0.67 | - | - | 0.33 |
| 21 | - | 0.67 | 0.67 | - | 0.67 | 0.67 | - | 0.67 | 0.67 | - | 0.67 | - | 0.67 |

This table shows a Rule-Based evaluation for Patient 7(PID7), based on the Rule base specified– using 21 rules. The table shows the final outcome of the Non-zero minimum values
