# Supplementary material for: MAVSCOT: A fuzzy logic-based HIV diagnostic system with indigenous multi-lingual interfaces for rural Africa
Source: PLoS One. 2020 Nov 6;15(11):e0241864. doi: 10.1371/journal.pone.0241864 (PMC7647102; doi:10.1371/journal.pone.0241864)
Supplement: S9 Table — This table shows the sets of rules that met the Non-zero minimum values criteria. These are: Rules 1, 4, 5, 6, 7, 8, 9, 10, 11, 16, 17, 18, 19, 20, 21. These can be respectively classified as follows: Mild = None; Moderate = R4, R5, R6, R8, R18, R19; Severe = R1, R7, R9, R10, R11, R16, R17, R19, R20, R21. (DOC) [file pone.0241864.s015.doc]

**S9 Table. List of Rules that produced non-zero minimum values.**

| Rule No. | Abnormal swelling | Anxiety | Dementia | Fatigue | Fever | Headache | Sexual dysfunction | Night sweats | Joint Pain (Rheumatism | Muscle aches | Ulcers in the Genitals | Weight loss | Non-Zero Mimimum Values and HIV severity |
| --- | --- | --- | --- | --- | --- | --- | --- | --- | --- | --- | --- | --- | --- |
| 1 | - | - | - | 0.67 | - | 0.67 | - | - | 0.67 | - | - | - | 0.67 Severe |
| 2 | - | - | - | - | - | - | - | - | - | - | - | 0 | Moderate |
| 3 | - | - | - | - | - | - | - | - | - | - | - | 0 | Mild |
| 4 | - | - | - | - | - | - | 0.67 | 0.67 | 0.67 | 0.67 | 0.67 | - | 0.67 Severe |
| 5 | - | 0.67 | - | 0.67 | - | 0.67 | - | 0.67 | - | - | - | 0 | 0.67 Severe |
| 6 | - | 0.67 | - | 0.67 | - | 0.67 | 0.67 | - | 0.67 | - | 0.67 | 0 | 0.67 Severe |
| 7 | 0.67 | - | - | - | 0.67 | - | 0.67 | - | 0.67 | 0.67 | 0.67 | - | 0.67 Severe |
| 8 | - | - | - | - | 0.67 | - | 0.67 | - | 0.67 | - | - | 0 | 0.67 Severe |
| 9 | - | - | 0.33 | - | - | - | - | - | - | - | - | - | 0.33 Mild |
| 10 | 0.67 | 0.67 | - | 0.67 | 0.67 | 0.67 | 0.67 | 0.67 | 0.67 | 0.67 | 0.67 | - | 0.67 Severe |
| 11 | - | - | 0.67 | 0.67 | - | - | 0.67 | 0.67 | - | - | 0.67 | 0 | 0.67 Severe |
| 12 | - | - | - | - | - | - | - | - | - | - | - | - | - Moderate |
| 13 | - | - | - | - | - | - | - | - | - | - | - | 0 | Mild |
| 14 | - | - | - | - | - | - | - | - | - | - | - | 0 | Mild |
| 15 | - | - | - | - | - | - | - | - | - | - | - | - | - Moderate |
| 16 | 0.67 | - | 0.33 | 0.67 | - | - | 0.67 | - | - | 0.67 | - | - | 0.33 Moderate |
| 17 | - | - | 0.33 | - | - | - | 0.67 | 0.67 | 0.67 | - | - | - | 0.33 Moderate |
| 18 | - | 0.67 | 0.33 | - | 0.67 | - | - | 0.67 | - | - | - | - | 0.33 Moderate |
| 19 | - | - | - | 0.67 | - | - | - | - | - | 0.67 | - | 0 | 0.67 Severe |
| 20 | 0.67 | - | 0.33 | 0.67 | - | - | 0.67 | - | - | 0.67 | - | - | 0.33 Moderate |
| 21 | - | 0.67 | 0.67 | - | 0.67 | 0.67 | - | 0.67 | 0.67 | - | 0.67 | - | 0.67 Severe |

This table shows the sets of rules that met the Non-zero minimum values criteria. These are: Rules 1,4,5,6, 7, 8, 9, 10, 11, 16, 17, 18, 19, 20, 21. These can be respectively classified as follows:

Mild = None

Moderate=R4, R5, R6, R8, R18, R19,

Severe =R1, R7, R9, R10, R11, R16, R17, R19, R20, R21,
