# Supplementary material for: MAVSCOT: A fuzzy logic-based HIV diagnostic system with indigenous multi-lingual interfaces for rural Africa
Source: PLoS One. 2020 Nov 6;15(11):e0241864. doi: 10.1371/journal.pone.0241864 (PMC7647102; doi:10.1371/journal.pone.0241864)
Supplement: S10 Table — This table shows the results produced by MAVSCOT in English, Afrikaans, IsiXhosa and Zulu languages. These are the percentage of overall HIV severity diagnosed per patient. (DOC) [file pone.0241864.s016.doc]

S10 Table: Predicted HIV diagnosis results by MAVSCOT for the female patient

| **HIV predicted results of the English Version of the Multilingual indigenous Informatics(MAVSCOT) Software** | **HIV predicted results of the Afrikaans Version of the Multilingual indigenous Informatics(MAVSCOT) Software** | **HIV predicted results of the IsiXhosa Version of the Multilingual indigenous Informatics(MAVSCOT) Software** | **HIV predicted results of the Zulu Version of the Multilingual indigenous Informatics(MAVSCOT) Software** |
| --- | --- | --- | --- |
| 68.04%68% HIV severity diagnosed | 68.04%68% HIV severity diagnosed | 68.04%68% HIV severity diagnosed | 68.24%68% HIV severity diagnosed. |

This table shows the results produced by MAVSCOT in English, Afrikaans, IsiXhosa and Zulu languages. These are the percentage of overall HIV severity diagnosed per patient.
