# Supplementary material for: MAVSCOT: A fuzzy logic-based HIV diagnostic system with indigenous multi-lingual interfaces for rural Africa
Source: PLoS One. 2020 Nov 6;15(11):e0241864. doi: 10.1371/journal.pone.0241864 (PMC7647102; doi:10.1371/journal.pone.0241864)
Supplement: S11 Table — This table provides a comparative analysis between MAVSCOT software and other existing HIV voice-enabled expert system/software. The metrics used for this comparison include the description, multi-lingual features (text-based), Voice-Enabled/Speech-based features, Functionalities—HIV Predictive Feature, Advisory Features, input and output data. (DOC) [file pone.0241864.s017.doc]

**S11 Table**: Comparison of existing HIV voice-enabled expert system/software with HIV multilingual indigenous informatics software

| References | Pazzani et al., 1997) | Tucker et al., 2013). | *Ebrahimi et al., 2013) | *Atalay et al., 1999), | Oluwagbemi et al., ) MAVSCOT |
| --- | --- | --- | --- | --- | --- |
| Comparative Features/Factors |
| 1. Description | Pazzani and colleagues, applied the knowledge of rule-based expert systems to the management of HIV-infected patients. Their system encodes information from existing literature of known drug resistant mutations. | Tucker and colleagues examined how a telephone-based IVR (Interactive Voice Response) self-monitoring system can be used to access daily HIV anti-retroviral medication adherence | Ebrahimi and colleagues developed an intelligent AIDS/HIV web-based medical consulting system which provides consulting services on systematic textual data  *Web-based application | Atalay and colleagues developed an interactive web-based HIV patient care expert systems | Multilingual Indigenous HIV Informatics Software for South Africa  *Standalone application |
| 2. Multilingual Features (Text-based) | Nil | Nil | Nil | Nil | Yes  Four(4) languages – English language and three(3) South African indigenous languages-Afrikaans, IsiXhosa and Zulu |
| 3.Voice-Enbled/Speech-based features | Nil | Yes  An Interactive Voice Response (IVR) system was developed. | Nil | Nil  Although interactive | Yes  *Not interactive but Voice-enabled system. *Pronunciation and Intonations in English language was good.  *Although the pronunciations and intonations of speech-features of Afrikaans, IsiXhosa and Zulu are not perfect. More work still need to be done. |
| 4. HIV Predictive Feature , Advisory Features (Functionalities) | Yes  *applied the knowledge of rule-based expert systems.  * System encodes information from existing literature of known HIV drug resistant mutations. | Nil | Nil | Nil  *it’s a web-based, question-answer session, patient-care system, based on inference engine and knowledge base | Yes  Fuzzy-logic-rule-based predictive mechanism  *it has inference engine for the fuzzy concepts and knowledge base to store rules and HIV symptoms |
| 5. Input Data | *information from the literature of known HIV drug resistance mutations | *Voice in English only | Textual data | Textual data | *HIV (textual data) symptoms to be selected  *Demographic textual data selection  *History of Health record data selection |
| 6. Output data | Textual data | *Voice in English only | *Textual data  *Provides answers to inquiries made online about HIV | Textual data | *Predicts HIV intensity (%) in numbers and in Voice/speech  *Prescribes HIV drugs with the corresponding dosage (text and voice/speech)  *Provides advice to patients(text and voice/speech)  *Provides recommendation to patients.(text and voice/speech) |

This table provides a comparative analysis between MAVSCOT software and other existing HIV voice-enabled expert system/software. The metrics used for this comparision include the description, multi-lingual features (text-based), Voice-Enbled/Speech-based features, Functionalities - HIV Predictive Feature , Advisory Features, input and output data.
