# Supplementary material for: MAVSCOT: A fuzzy logic-based HIV diagnostic system with indigenous multi-lingual interfaces for rural Africa
Source: PLoS One. 2020 Nov 6;15(11):e0241864. doi: 10.1371/journal.pone.0241864 (PMC7647102; doi:10.1371/journal.pone.0241864)
Supplement: S12 Table — This table is the Fuzzy Rule-Base table for a second HIV patient. This table consists of an expanded number of HIV symptoms (24 HIV symptoms), with 14 rules. The table also provided answers to some of the lifestyle questions in MAVSCOT, before determining the diagnosis of the severity of the HIV of the second patient. Most of the results in this table, show that the HIV diagnosis is SEVERE unlike the table for the first HIV patient that had most of the diagnosed cases of HIV diagnosis as MODERATE. (DOC) [file pone.0241864.s018.doc]

S12 Table: Fuzzy Rule Base for the HIV Multilingual Informatics Software in English and three (3) indigenous South African languages – Using 14 Rules

|  | Symptoms |  |  |  |  |  |  |  |  |  |  |  |  |  |  |  |  |  |  |  |  |  |  |  | Q1 | Q2 | Q3 | Q4 | Q5 | Q6 | Conclusio[Prediction] |  |
| --- | --- | --- | --- | --- | --- | --- | --- | --- | --- | --- | --- | --- | --- | --- | --- | --- | --- | --- | --- | --- | --- | --- | --- | --- | --- | --- | --- | --- | --- | --- | --- | --- |
| Rule Number | Weight Loss  [S39] | Vomitting[S38] | Ulcer on the Genitals[S36] | Swollen Lymph Nodes[S35] | Stomach Upset[S34] | Soreness of the Vagina[S33] | Sexual Dysfunction[S30] | Painful Urination[S28] | Painful Intercourse[S27 | Pain U.R. Abdomen[S26 | Missed periods[S21] | Lower Abdominal Pain[S19] | Joint Pain[S18 | Itching in the Vaginal Area[S17 | Heavier or Lighter Periods[S16 | Gonorrhoea[S14] | Forgetfulness[S13] | Depression[S9 | Diarrhoea[S10 | Dementia(Memory Loss  [S8 | Body Temperature[S5] | Anxiety[S3] | Abnormal vaginal discharge[S2] | Abdominal Swelling[S1] |  |  |  |  |  |  |  |  |
| 1 | Mild | Moderate | Severe | Severe | Moderate | Severe | Moderate | Moderate | Moderate | Mild | Moderate | Severe | Severe | Moderate | Severe | Moderate | Moderate | Moderate | Mild | Moderate | Severe | Severe | Moderate | Severe | Y | Y | Y | Y | N | Y | English[62.19%];  Afrikaans[62.19%]  IsiXhosa[62.19%]  Zulu [62.19%] | SEVERE |
| 2 | Moderate | Moderate | Mild | Mild | Mild | Mild | Mild | Mild | Mild | Moderate | Moderate | Mild | Mild | Mild | Mild | Mild | Mild | Mild | Moderate | Moderate | Mild | Mild | Mild | Mild | N | Y | Y | Y | N | Y | English  [45.91%]  Afrikaans[45.91%  IsiXhosa[45.91%  Zulu [45.91% | MODERATE |
| 3 | Mild | Mild | Mild | Mild | Mild | Mild | Mild | Mild | Mild | Mild | Mild | Mild | Mild | Mild | Mild | Mild | Mild | Mild | Mild | Mild | Mild | Mild | Mild | Mild | Y | Y | Y | Y | Y | N | English  [17.69%]  Afrikaans[17.69%]  IsiXhosa[[17.69%]  Zulu [17.69%] | MILD |
| 4 | Moderate | Mild | Mild | Mild | Mild | Mild | Severe | Severe | Severe | Moderate | Mild | Mild | Mild | Mild | Mild | Severe | Severe | Severe | Moderate | Mild | Mild | Mild | Mild | Mild | N | N | Y | Y | Y | N | English  [52.17%]  Afrikaans[52.17%]  IsiXhosa[[52.17%]  Zulu [52.17%] | SEVERE |
| 5 | Mild | Mild | Mild | Mild | Severe | Moderate | Severe | Mild | Mild | Mild | Mild | Mild | Mild | Severe | Moderate | Severe | Mild | Mild | Mild | Mild | Mild | Mild | Severe | Moderate | N | N | N | N | N | Y | English  [47.31%]  Afrikaans[47.31%]  IsiXhosa[[47.31%]  Zulu [47.31%] | MODERATE |
| 6 | Mild | Severe | Mild | Severe | Mild | Severe | Mild | Mild | Mild | Mild | Severe | Mild | Severe | Mild | Severe | Mild | Mild | Mild | Mild | Severe | Mild | Severe | Mild | Severe | Y | N | Y | N | Y | N | English  [53.42%]  Afrikaans[53.42%]  IsiXhosa[[53.42%]  Zulu [53.42%] | SEVERE |
| 7 | Severe | Mild | Severe | Mild | Severe | Mild | Severe | Mild | Severe | Severe | Mild | Severe | Mild | Severe | Mild | Severe | Mild | Severe | Severe | Mild | Severe | Mild | Severe | Mild | N | Y | N | Y | N | Y | English  [57.52%]  Afrikaans[57.52%]  IsiXhosa[[57.52%]  Zulu [57.52%] | SEVERE |
| 8 | Mild | Mild | Mild | Mild | Severe | Mild | Severe | Mild | Mild | Mild | Mild | Mild | Mild | Severe | Mild | Severe | Mild | Mild | Mild | Mild | Mild | Mild | Severe | Mild | N | N | N | Y | Y | Y | English  [46.15%]  Afrikaans[46.15%]  IsiXhosa[[46.15%]  Zulu [46.15%] | MODERATE |
| 9 | Moderate | Moderate | Moderate | Moderate | Moderate | Moderate | Moderate | Moderate | Moderate | Moderate | Moderate | Moderate | Moderate | Moderate | Moderate | Moderate | Moderate | Moderate | Moderate | Moderate | Moderate | Moderate | Moderate | Moderate | N | N | N | N | Y | Y | English  [56.44%]  Afrikaans[56.44%]  IsiXhosa[[56.44%]  Zulu [56.44%] | SEVERE |
| 10 | Severe | Severe | Severe | Severe | Severe | Severe | Severe | Severe | Severe | Severe | Severe | Severe | Severe | Severe | Severe | Severe | Severe | Severe | Severe | Severe | Severe | Severe | Severe | Severe | Y | Y | Y | Y | Y | Y | English  [73.08%]  Afrikaans[73.08%]  IsiXhosa[[73.08%]  Zulu [73.08%] | SEVERE |
| 11 | Mild | Mild | Severe | Severe | Mild | Mild | Severe | Severe | Mild | Mild | Severe | Severe | Mild | Mild | Severe | Severe | Mild | Mild | Severe | Severe | Mild | Mild | Severe | Severe | N | N | N | N | N | N | English  [57.24%]  Afrikaans[57.24%]  IsiXhosa[[57.24%]  Zulu [57.24%] | SEVERE |
| 12 | Severe | Severe | Severe | Severe | Severe | Severe | Severe | Severe | Severe | Severe | Severe | Severe | Severe | Severe | Severe | Severe | Severe | Severe | Severe | Severe | Severe | Severe | Severe | Severe | N | N | N | N | N | N | English  [72.64%]  Afrikaans[72.64%]  IsiXhosa[[72.64%]Zulu [72.64%] | SEVERE |
| 13 | Mild | Mild | Mild | Moderate | Moderate | Moderate | Moderate | Moderate | Severe | Mild | Mild | Mild | Moderate | Moderate | Moderate | Moderate | Moderate | Severe | Mild | Mild | Mild | Moderate | Moderate | Moderate | Y | Y | N | N | Y | Y | English  [53.55%]  Afrikaans[53.55%]  IsiXhosa[[53.55%]Zulu [53.55%] | SEVERE |
| 14 | Severe | Severe | Severe | Severe | Severe | Moderate | Moderate | Severe | Severe | Severe | Severe | Severe | Severe | Severe | Moderate | Moderate | Severe | Severe | Severe | Severe | Severe | Severe | Severe | Moderate | N | N | N | Y | N | Y | English  [71.59%]  Afrikaans[71.59%]  IsiXhosa[[71.59%]Zulu [71.59%] | SEVERE |

This table is the Fuzzy Rule-Base table for a second HIV patient. This table consists of an expanded number of HIV symptoms (24 HIV symptoms), with 14 rules. The table also provided answers to some of the lifestyle questions in MAVSCOT, before determining the diagnosis of the severity of the HIV of the second patient. Most of the results in this table, show that the HIV diagnosis is SEVERE unlike the table for the first HIV patient that had most of the diagnosed cases of HIV diagnosis as MODERATE.
