# Supplementary material for: MAVSCOT: A fuzzy logic-based HIV diagnostic system with indigenous multi-lingual interfaces for rural Africa
Source: PLoS One. 2020 Nov 6;15(11):e0241864. doi: 10.1371/journal.pone.0241864 (PMC7647102; doi:10.1371/journal.pone.0241864)
Supplement: S13 Table — This table provides information about the 24 HIV symptoms of a second patient used as illustration for the demonstration of the diagnosis of the MAVSCOT software. This table consists of the rating on the variables, and the triangular fuzzification function values. (DOC) [file pone.0241864.s019.doc]

**S13 Table: Example of Symptoms, Severity, Rating on Variables of 24 HIV Symptoms of another patient**

| **HIV Symptoms Code** | **HIV Symptoms of HIV patients obtained from Medical and Scientific literature** | **Severity of HIV infections** | **Rating on Variables** | **Triangular Fuzzification Function values** |
| --- | --- | --- | --- | --- |
| S39 | Weight Loss | Severe | 3 | ((3-1)/3) = 0.67 |
| S38 | Vomiting | Severe | 3 | ((3-1)/3) = 0.67 |
| S36 | Ulcer at Genitals | Severe | 3 | ((3-1)/3) = 0.67 |
| S35 | Swollen Lymph Nodes | Severe | 3 | ((3-1)/3) = 0.67 |
| S34 | Stomach Upset | Severe | 3 | ((3-1)/3) = 0.67 |
| S33 | Soreness of the Vagina | Moderate | 2 | ((3-2)/3) = 0.33 |
| S30 | Sexual Dysfunction | Severe | 3 | ((3-1)/3) = 0.67 |
| S28 | Painful Urination | Severe | 3 | ((3-1)/3) = 0.67 |
| S27 | Painful Intercourse | Severe | 3 | ((3-1)/3) = 0.67 |
| S26 | Pain in the Upper right abdomen | Severe | 3 | ((3-1)/3) = 0.67 |
| S21 | Missed Periods | Severe | 3 | ((3-1)/3) = 0.67 |
| S19 | Lower Abdominal pain | Mild | 1 | ((1-1)/3) = 0 |
| S18 | Joint Pain (Rheumatism) | Severe | 3 | ((3-1)/3) = 0.67 |
| S17 | Itching in the Vagina area | Severe | 3 | ((3-1)/3) = 0.67 |
| S16 | Heavier or Lighter Periods | Severe | 3 | ((3-1)/3) = 0.67 |
| S14 | Gonorrhoea | Severe | 3 | ((3-1)/3) = 0.67 |
| S13 | Forgetfulness | Moderate | 2 | ((3-2)/3) = 0.33 |
| S9 | Depression | Severe | 3 | ((3-1)/3) = 0.67 |
| S10 | Diarrhoea | Severe | 3 | ((3-1)/3) = 0.67 |
| S8 | Dementia (MemoryLoss) | Severe | 3 | ((3-1)/3) = 0.67 |
| S5 | Body Temperature | Severe | 3 | ((3-1)/3) = 0.67 |
| S3 | Anxiety | Mild | 1 | ((1-1)/3) = 0 |
| S2 | Abnormal Vagina discharge | Severe | 3 | ((3-1)/3) = 0.67 |
| S1 | Abdominal Swelling | Severe | 3 | ((3-1)/3) = 0.67 |

This table provides information about the 24 HIV symptoms of a second patient used as illustration for the demonstration of the diagnosis of the MAVSCOT software. This table consists of the rating on the variables, and the triangular fuzzification function values.
