# Supplementary material for: MAVSCOT: A fuzzy logic-based HIV diagnostic system with indigenous multi-lingual interfaces for rural Africa
Source: PLoS One. 2020 Nov 6;15(11):e0241864. doi: 10.1371/journal.pone.0241864 (PMC7647102; doi:10.1371/journal.pone.0241864)
Supplement: S14 Table — This table shows the Triangular Fuzzy Function Values. (DOC) [file pone.0241864.s020.doc]

**S14 Table:** Triangular Fuzzy Function Values

| **Symptoms Codes** | S39 | S38 | S36 | S35 | S34 | S33 | S30 | S28 | S27 | S26 | S21 | S19 | S18 | S17 | S16 | S14 | S13 | S9 | S10 | S8 | S5 | S3 | S2 | S1 |
| --- | --- | --- | --- | --- | --- | --- | --- | --- | --- | --- | --- | --- | --- | --- | --- | --- | --- | --- | --- | --- | --- | --- | --- | --- |
| **Triangular Fuzzy Function**  **Values** | 0.67 | 0.67 | 0.67 | 0.67 | 0.67 | 0.33 | 0.67 | 0.67 | 0.67 | 0.67 | 0.67 | 0 | 0.67 | 0.67 | 0.67 | 0.67 | 0.33 | 0.67 | 0.67 | 0.67 | 0.67 | 0 | 0.67 | 0.67 |

This table shows the Triangular Fuzzy Function Values
