# Supplementary material for: MAVSCOT: A fuzzy logic-based HIV diagnostic system with indigenous multi-lingual interfaces for rural Africa
Source: PLoS One. 2020 Nov 6;15(11):e0241864. doi: 10.1371/journal.pone.0241864 (PMC7647102; doi:10.1371/journal.pone.0241864)
Supplement: S15 Table — This table shows the 24 HIV symptoms, and how the Fuzzy Rule-base values have been able to produce HIV diagnosis and their non-zero minimum values. (DOC) [file pone.0241864.s021.doc]

**S15 Table: Table of 24 HIV Symptoms, Non-zero Minimum Values and HIV diagnosis**

This table shows the 24 HIV symptoms, and how the Fuzzy Rule-base values have been able to produce HIV diagnosis and their non-zero minimum values

|  | Symptoms |  |  |  |  |  |  |  |  |  |  |  |  |  |  |  |  |  |  |  |  |  |  |  | Conclusio[Prediction] | Non Zero Minimum Value |
| --- | --- | --- | --- | --- | --- | --- | --- | --- | --- | --- | --- | --- | --- | --- | --- | --- | --- | --- | --- | --- | --- | --- | --- | --- | --- | --- |
| Rule Number (R ) | Weight Loss  [S39] | Vomitting[S38] | Ulcer on the Genitals[S36] | Swollen Lymph Nodes[S35] | Stomach Upset[S34] | Soreness of the Vagina[S33] | Sexual Dysfunction[S30] | Painful Urination[S28] | Painful Intercourse[S27 | Pain U.R. Abdomen[S26 | Missed periods[S21] | Lower Abdominal Pain[S19] | Joint Pain[S18 | Itching in the Vaginal Area[S17 | Heavier or Lighter Periods[S16 | Gonorrhoea[S14] | Forgetfulness[S13] | Depression[S9 | Diarrhoea[S10 | Dementia(Memory Loss  [S8 | Body Temperature[S5] | Anxiety[S3] | Abnormal vaginal discharge[S2] | Abdominal Swelling[S1] |  |  |
| 1 | - | 0.67 | 0.67 | - | - | - | - | - | - | - | - | - | 0.67 | - | 0.67 | - | 0.33 | - | - | - | 0.67 | - | - | 0.67 | Moderate | 0.33 |
| 2 | - | - | - | - | - | - | - | - | - | - | - | 0 | - | - | - | - | - | - | - | - | - | 0 | - | - | Mild | 0 |
| 3 | - | - | - | - | - | - | - | - | - | - | - | 0 | - | - | - | - | - | - | - | - | - | 0 | - | - | Mild | 0 |
| 4 | - | - | - | - | - | - | 0.67 | 0.67 | 0.67 | - | - | - | - | - | - | 0.67 | - | 0.67 | - | - | - | - | - | - | Severe | 0.67 |
| 5 | - | - | - | - | 0.67 | 0.33 | 0.67 | - | - | - | - | 0 | - | 0.67 | - | 0.67 | - | - |  | - | - | 0 | 0.67 | - | Moderate | 0.33 |
| 6 |  | 0.67 |  | 0.67 |  |  |  | 0.67 |  | 0.67 |  | 0 |  | 0.67 |  | 0.67 |  | 0.67 |  | 0.67 | - | - | - | 0.67 | Severe | 0.67 |
| 7 | 0.67 | - | 0.67 | - | 0.67 | - | 0.67 | - | 0.67 | 0.67 | - | - | - | 0.67 | - | 0.67 | - | 0.67 | 0.67 | - | 0.67 | 0 | 0.67 | - | Severe | 0.67 |
| 8 | - | - | - | - | 0.67 | - | - | - | - | - | 0 | - | 0.67 | - | 0.67 | - | - | - | - | - | - | 0 | 0.67 | - | Severe | 0.67 |
| 9 | - | - | - | - | - | 0.33 | - | - | - | - | - | - | - | - | - | - | 0.33 | - | - | - | - | - | - | - | Moderate | 0.33 |
| 10 | 0.67 | 0.67 | 0.67 | 0.67 | 0.67 | - | 0.67 | 0.67 | 0.67 | 0.67 | 0.67 | - | 0.67 | 0.67 | 0.67 | 0.67 | - | 0.67 | 0.67 | 0.67 | 0.67 | - | 0.67 | 0.67 | Severe | 0.67 |
| 11 | - | - | 0.67 | 0.67 | - | - | 0.67 | 0.67 | - | - | 0.67 | - | - | - | 0.67 | 0.67 | - | - | 0.67 | 0.67 | - | 0 | 0.67 | 0.67 | Severe | 0.67 |
| 12 | 0.67 | 0.67 | 0.67 | 0.67 | 0.67 | - | 0.67 | 0.67 | 0.67 | 0.67 | 0.67 | - | 0.67 | 0.67 | 0.67 | 0.67 | - | 0.67 | 0.67 | 0.67 | 0.67 | - | 0.67 | 0.67 | Severe | 0.67 |
| 13 | - | - | - | - | - | 0.33 | - | - | 0.67 | - | - | 0 | - | - | - | - | 0.33 | 0.67 | - | - | - | - | - | - | Moderate | 0.33 |
| 14 | 0.67 | 0.67 | 0.67 | 0.67 | 0.67 | 0.33 | - | 0.67 | 0.67 | 0.67 | - | 0.67 | 0.67 | 0.67 | - | - | - | 0.67 | 0.67 | 0.67 | 0.67 | - | 0.67 | - | Moderate | 0.33 |
