# Supplementary material for: MAVSCOT: A fuzzy logic-based HIV diagnostic system with indigenous multi-lingual interfaces for rural Africa
Source: PLoS One. 2020 Nov 6;15(11):e0241864. doi: 10.1371/journal.pone.0241864 (PMC7647102; doi:10.1371/journal.pone.0241864)
Supplement: S2 Appendix — This file contains all figures used in the main manuscript and supporting documents. (DOC) [file pone.0241864.s023.doc]

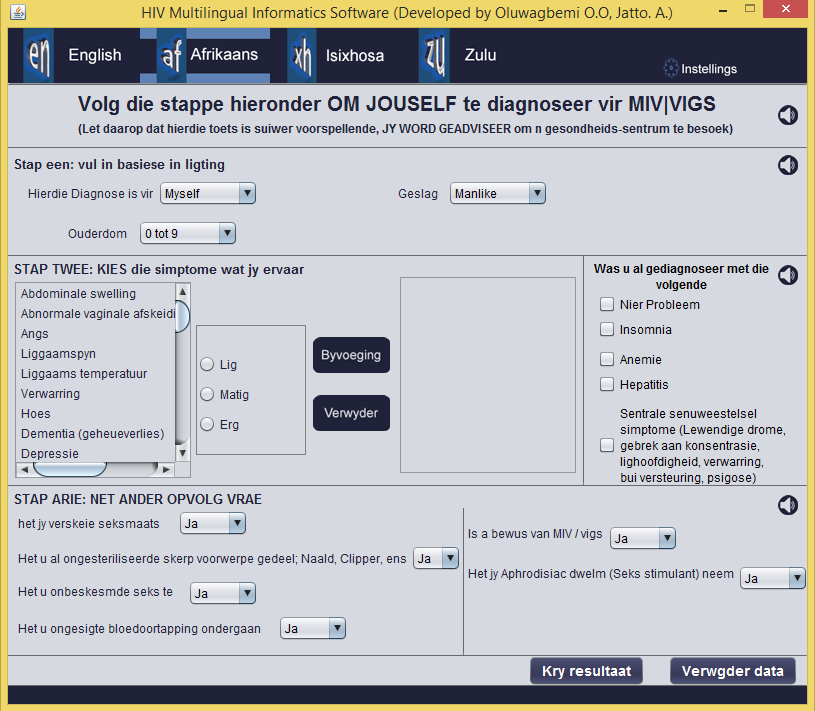


**S4 Figure.** The Afrikaans language Graphical User Interface of the multi-lingual HIV indigenous fuzzy-logic diagnostic system (MAVSCOT). This GUI provides the description of the various segments/sections of the interface in the Afrikaans language. The Afrikaans GUI Is also a multilingual HIV voice-enabled software (specifically in Afrikaans language).


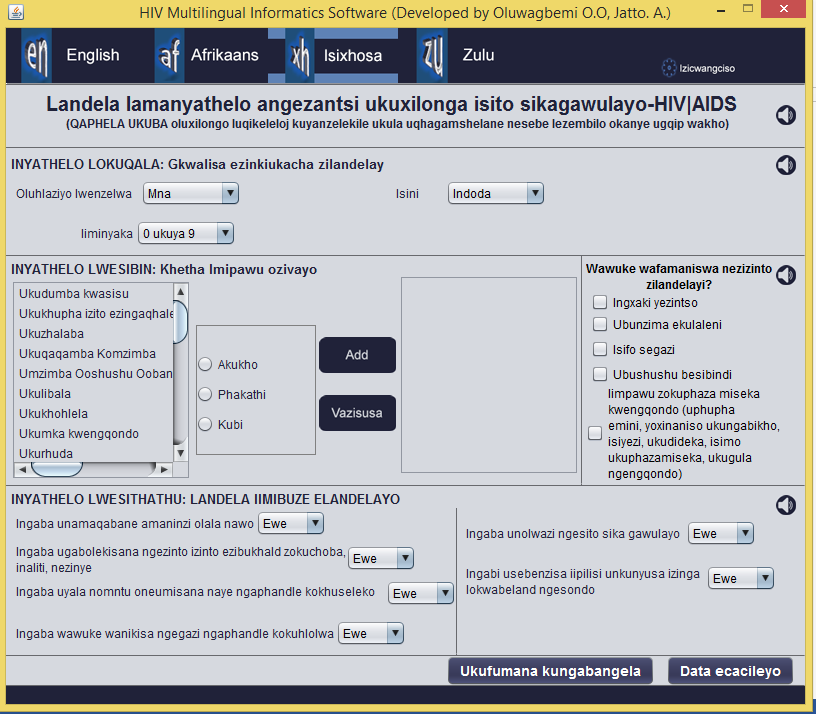
1

**Figure S5.** The Graphical User Interface of the multi-lingual HIV indigenous fuzzy-logic diagnostic system (MAVSCOT) for IsiXhosa language. The system is also a multilingual HIV voice-enabled software.

**S5 Figure.** The **IsiXhosa** language Graphical User Interface of the multi-lingual HIV indigenous fuzzy-logic diagnostic system (MAVSCOT). This GUI provides the description of the various segments/sections of the interface in the **IsiXhosa** language. The **IsiXhosa** GUI Is also a multilingual HIV voice-enabled software (specifically in **IsiXhosa** language).

**
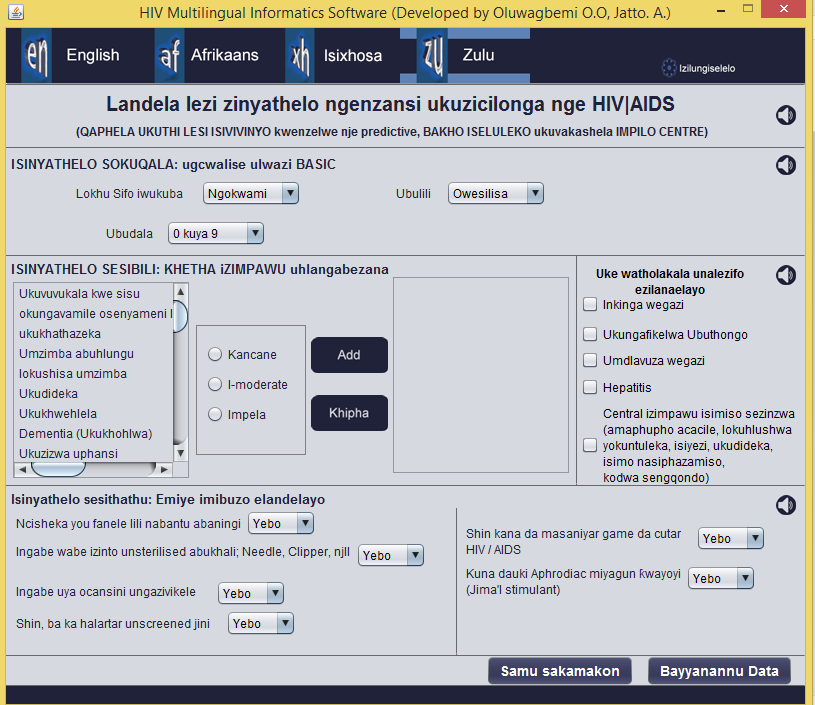
**

1

**Figure S6.** The Graphical User Interface (GUI) of the multi-lingual HIV indigenous fuzzy-logic diagnostic system (MAVSCOT) for Zulu language. The system is also a multilingual HIV voice-enabled software.

**S6 Figure.** The **Zulu** language Graphical User Interface of the multi-lingual HIV indigenous fuzzy-logic diagnostic system (MAVSCOT). This GUI provides the description of the various segments/sections of the interface in the **Zulu** language. The **Zulu** GUI Is also a multilingual HIV voice-enabled software (specifically in **Zulu** language).

Results – Supplementary material


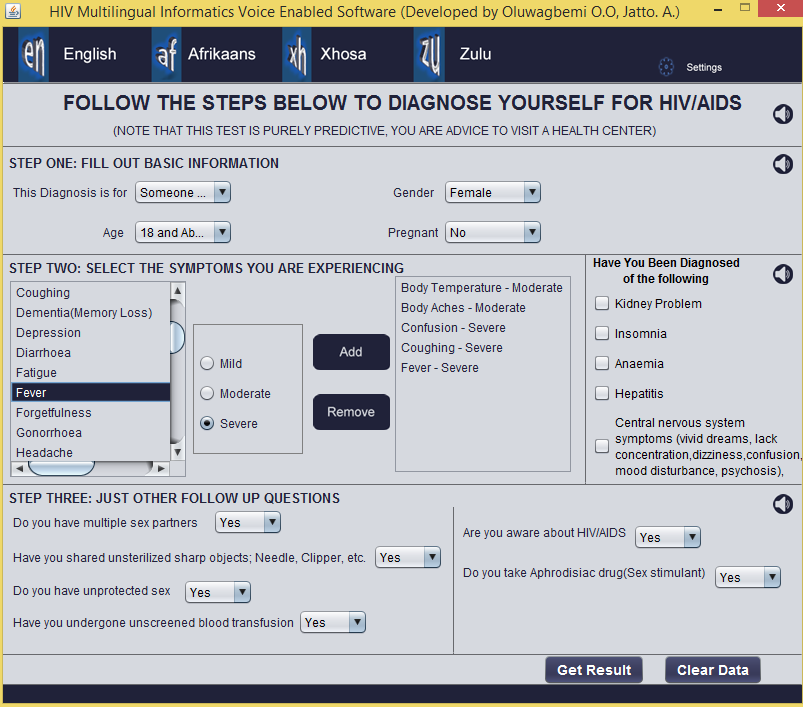


**Figure S7.** The Graphical User Interface of the HIV Informatics Multi-lingual Voice Enabled Software (MAVSCOT) for English language showing HIV Symptoms keyed-in in English Language

**S7 Figure.** The **English** language Graphical User Interface of the multi-lingual HIV indigenous fuzzy-logic diagnostic system (MAVSCOT), showing selected HIV symptoms for a patient. This GUI provides the description of the various segments/sections of the interface in the **English** language. The **English** GUI is also a multilingual HIV voice-enabled software (specifically in English language).

**
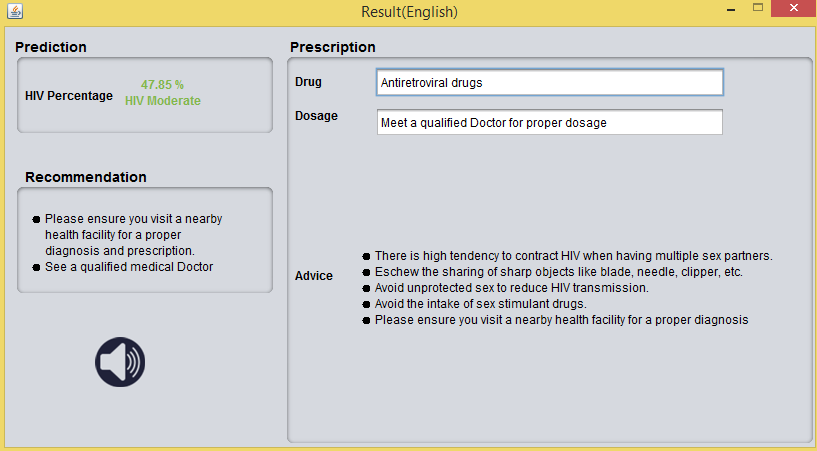
**

**Figure S8.** The Graphical User Interface of the HIV Informatics Multi-lingual Voice Enabled Software (MAVSCOT) for English language showing HIV diagnosis results

**S8 Fig. Result of English language Graphical User Interface of the multi-lingual HIV indigenous fuzzy-logic diagnostic system (MAVSCOT).** This results reveal the diagnosis result as ((47.85 HIV moderate) for the previously selected HIV symptoms for a patient. Other sections of this result are as follows: the prescription, recommendation and advice. This is the Fig S8 legend.


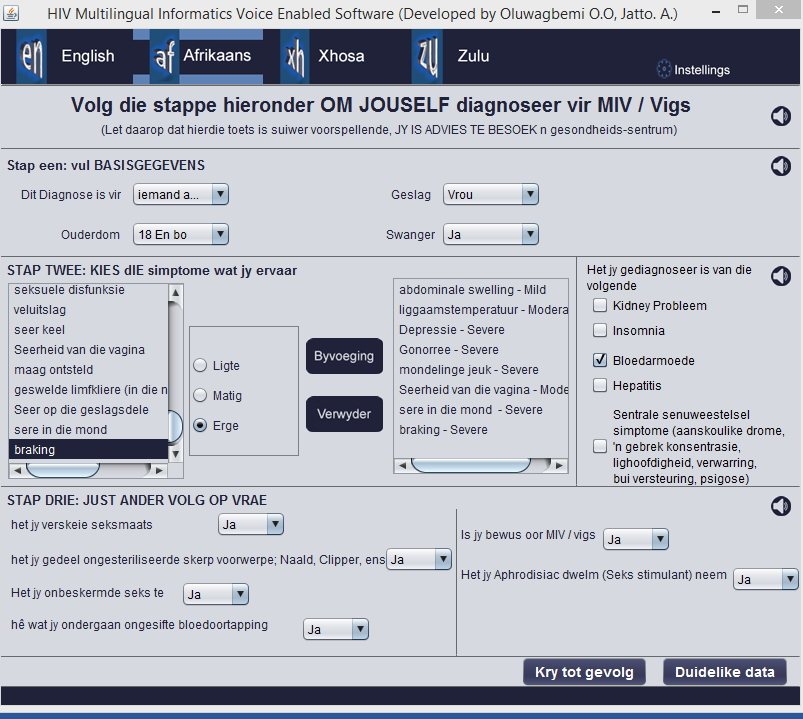


**Figure S9.** The Graphical User Interface of the HIV Informatics Multi-lingual Voice Enabled Software (MAVSCOT) showing HIV symptoms keyed-in in Afrikaans language.

**S9 Figure.** The **Afrikaans** language Graphical User Interface of the multi-lingual HIV indigenous fuzzy-logic diagnostic system (MAVSCOT), showing selected HIV symptoms for a patient. This GUI provides the description of the various segments/sections of the interface in the **Afrikaans** language. The **Afrikaans** GUI is also a multilingual HIV voice-enabled software (specifically in Afrikaans language). The HIV symptoms are pronounced in Afrikaans language.


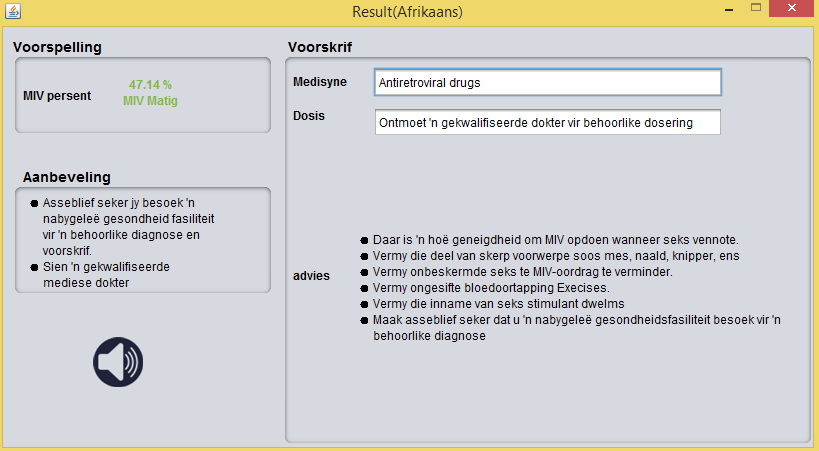


**Figure S10.** The Graphical User Interface of the HIV Informatics Multi-lingual Voice Enabled Software (MAVSCOT) showing Predicted Results in Afrikaans Language, for selected HIV symptoms.

**S10 Fig. Result of Afrikaans language Graphical User Interface of the multi-lingual HIV indigenous fuzzy-logic diagnostic system (MAVSCOT).** This results reveal the diagnosis result as ((47.14 HIV moderate) for the previously selected HIV symptoms for a patient. Other sections of this result are as follows: the prescription, recommendation and advice. This is the Fig S8 legend.


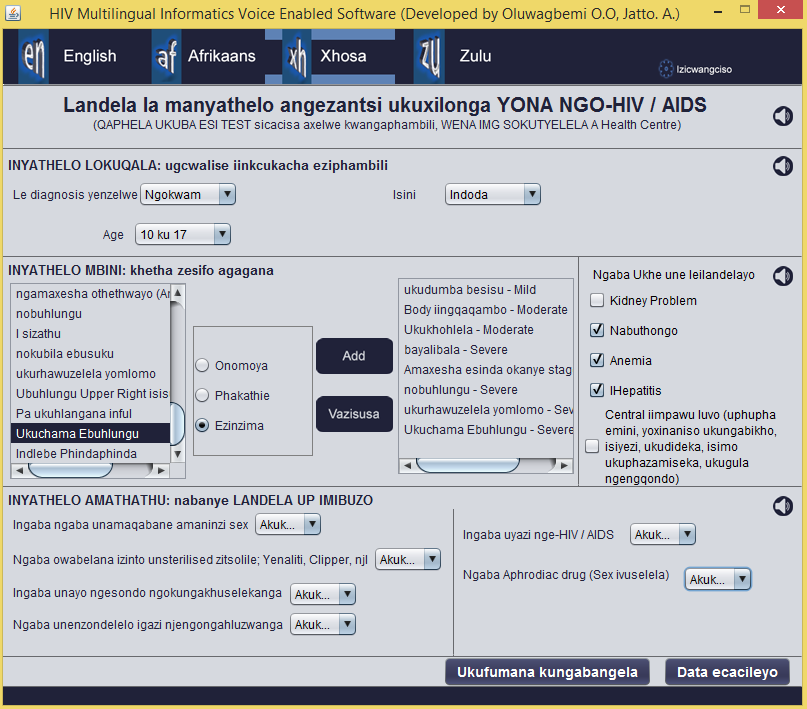
HIV Symptoms keyed-in in Xhosa Language

**Figure S11.** The Graphical User Interface of the HIV Informatics Multi-lingual Voice Enabled Software (MAVSCOT) showing HIV symptoms keyed-in in Xhosa language.

**S11 Figure.** The **IsiXhosa** language Graphical User Interface of the multi-lingual HIV indigenous fuzzy-logic diagnostic system (MAVSCOT), showing selected HIV symptoms for a patient. This GUI provides the description of the various segments/sections of the interface in the **IsiXhosa** language. The **IsiXhosa** GUI is also a multilingual HIV voice-enabled software (specifically in Afrikaans language). The HIV symptoms are pronounced in IsiXhosa language.


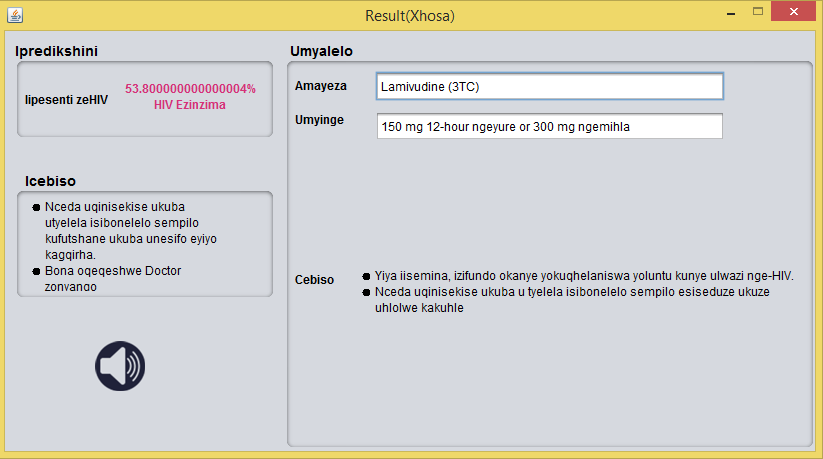


**Figure S12.** The Graphical User Interface of the HIV Informatics Multi-lingual Voice Enabled Software (MAVSCOT) showing Predicted Results in Xhosa Language, for selected HIV symptoms.

**S12 Fig. Result of IsiXhosa language Graphical User Interface of the multi-lingual HIV indigenous fuzzy-logic diagnostic system (MAVSCOT).** This results reveal the diagnosis result as ((53.80 HIV moderate) for the previously selected HIV symptoms for a patient. Other sections of this result are as follows: the prescription, recommendation and advice.


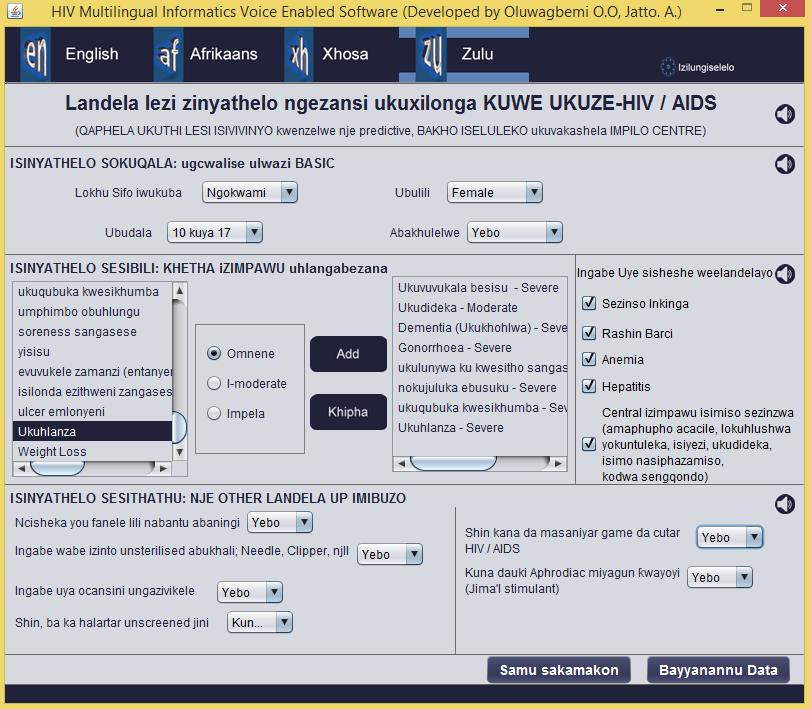
HIV Symptoms keyed-in in Zulu Language

**Figure S13.** The Graphical User Interface of the HIV Informatics Multi-lingual Voice Enabled Software (MAVSCOT) showing HIV symptoms keyed-in in Zulu language.

**S13 Figure.** The **Zulu** language Graphical User Interface of the multi-lingual HIV indigenous fuzzy-logic diagnostic system (MAVSCOT), showing selected HIV symptoms for a patient. This GUI provides the description of the various segments/sections of the interface in the **Zulu** language. The **Zulu** GUI is also a multilingual HIV voice-enabled software (specifically in Afrikaans language). The HIV symptoms are pronounced in Zulu language

Predicted Results in Zulu Language, for selected HIV symptoms


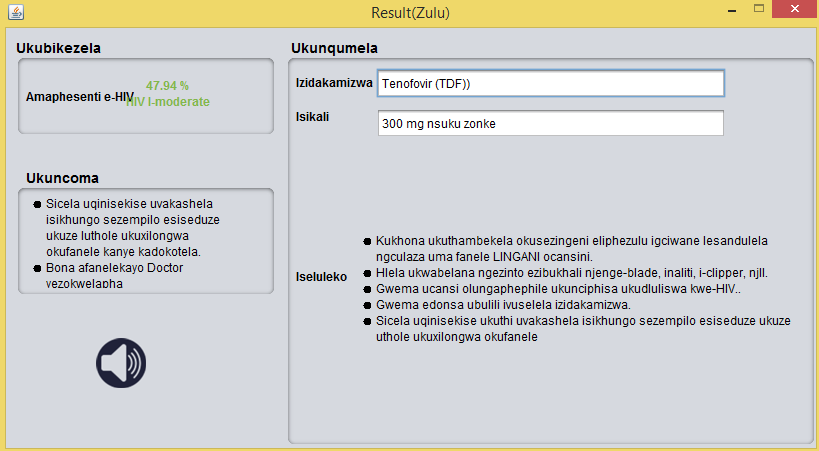


**Figure S14.** The Graphical User Interface of the HIV Informatics Multi-lingual Voice Enabled Software (MAVSCOT) showing Predicted Results in Zulu Language, for selected HIV symptoms

**S14 Fig. Result of Zulu language Graphical User Interface of the multi-lingual HIV indigenous fuzzy-logic diagnostic system (MAVSCOT).** This results reveal the diagnosis result as ((47.94 HIV moderate) for the previously selected HIV symptoms for a patient. Other sections of this result are as follows: the prescription, recommendation and advice.
