# Supplementary material for: MAVSCOT: A fuzzy logic-based HIV diagnostic system with indigenous multi-lingual interfaces for rural Africa
Source: PLoS One. 2020 Nov 6;15(11):e0241864. doi: 10.1371/journal.pone.0241864 (PMC7647102; doi:10.1371/journal.pone.0241864)
Supplement: S3 Appendix — This file contains an extra example to illustrate the implementation of fuzzy rule within the MAVSCOT software. (DOC) [file pone.0241864.s024.doc]

S3 Appendix

Another example to illustrate the implementation of fuzzy rule within the MAVSCOT software is presented here and applied to an experimental sample on HIV diagnoses within MAVSCOT.

The HIV symptoms of a female HIV patient, was keyed into the MAVSCOT software. These HIV symptoms were obtained from scientific literature (See Table S1). The algorithm for the MAVSCOT software was implemented and executed.

Second, the theory of fuzzy rule, fuzzy logic and fuzzy sets was implemented for the HIV symptoms of the female patient.

Third, the predicted diagnosis result from the MAVSCOT software was produced.

The subsequent sections provide a comprehensive description of this process:

We demonstrated this process by considering an example of a female HIV patient. We obtained 24 HIV symptoms (Weight Loss, Vomiting, Ulcer on the Genitals, Swollen Lymph Nodes, Stomach Upset, Soreness of the Vagina, Sexual Dysfunction, Painful Urination, Painful Intercourse, Pain in the Abdomen, Missed periods, Lower Abdominal Pain, Joint Pain, Itching in the Vaginal Area, Heavier or Lighter Periods, Gonorrhea, Forgetfulness, Depression, Dementia, Diarrhea, Body Temperature, Anxiety, Abnormal vaginal discharge, Abdominal swellings ), from scientific literature for the female patient (See Table S1 and Table S13). These HIV symptoms were keyed into the MAVSCOT software.

The MAVSCOT Algorithm was applied and the process is described below:

Step 1: HIV symptoms of the female HIV patient were extracted from medical and scientific literature [See Table S1.]. These HIV symptoms were re-coded into symbols, and tabulated into a new table showing the symbols, HIV symptoms, severity of the symptoms, and rating of the variables and generation of a Triangular fuzzy function values [See Table S14].

Step 2: Table S13 was used to generate the Triangular Fuzzy function values. See Table S13 and Table S14.

Step 3: Sample diagnosis was conducted by inputting the HIV symptoms into the MAVSCOT software under different forms of Fuzzy Rules (Rule 1 to Rule 14) and under different severity, coupled with corresponding possible behavioural lifestyles of patients. All these were captured and specified within the MAVSCOT software.

This process was carried out for the MAVSCOT English software, MAVSCOT Afrikaans software, MAVSCOT Xhosa (IsiXhosa) software, and the MAVSCOT Zulu software.

The predicted results for each of the software module were recorded in the Fuzzy Rule Base table. See Table S12.

Step 4: From Table S12, a new table was generated [See Table S15] depicting the rules that generated non-zero minimum values.

Step 5: The Rules that produced non-minimum zero values for different severity of HIV symptoms was extracted from Table S12.

So from Table S15,

For Mild cases of the HIV symptoms of the female patient, we have that:

Mild **= $\sqrt{R_{2}^{2}+R_{3}^{2}}$ = $\sqrt{0^{2}+0^{2}}$=** $\sqrt{0^{2}=}$0

For the Moderate cases of the HIV symptoms of the female patient, we have that:

Moderate = $\sqrt{R_{1}^{2}+R_{5}^{2}+R_{9}^{2}+R_{13}^{2}+R_{14}^{2}}=$ $\sqrt{0.33^{2}+0.33^{2}+0.33^{2}+0.33^{2}+0.33^{2}}=$ $\sqrt{0.1089+0.1089+0.1089+0.1089+0.1089}=$

$\sqrt{0.5445}=$0.7379

For the Severe cases of the HIV symptoms of the new female patient, we have that

Severe **=** $\sqrt{R_{4}^{2}+R_{6}^{2}+R_{7}^{2}+R_{8}^{2}+R_{10}^{2}+R_{11}^{2}+R_{12}^{2}}=$ $\sqrt{0.67^{2}+0.67^{2}+0.67^{2}+0.67^{2}+0.67^{2}+0.67^{2}+0.67^{2}}=$ $\sqrt{0.4489+0.4489+0.4489+0.4489+0.4489+0.4489+0.4489}=$

$\sqrt{3.1423}=$1.77265 = 1.7727

Step 6: In this step, the Center of Gravity (CoG) Technique was applied and defuzzification process takes place as follows:

Mild **=** $\frac{Mild(valueobtainedinStep5)}{NumberofRules}=$ $\frac{0}{2}=$0

Moderate **=** $\frac{Moderate(valueobtainedinStep5)}{NumberofRules}=$ $\frac{0.7379}{5}=0.1476$

Severe **=** $\frac{Severe(valueobtainedinStep5)}{NumberofRules}=$ $\frac{1.7727}{7}=0.2532$

Step 7: The fuzzy set is defuzzified into precise outputs. Applying the CoG method, defuzzification goes as follows:

Output = $\frac{(0X0.2)+(0.1476X0.45)+(0.2532X0.8)}{0+0.1476+0.2532}=$ $\frac{(0+0.06642+0.20256)}{0.4008}=$ $\frac{0.26898}{0.4008}=$ $\frac{0.27}{0.4}X100=$67.5%

Output = 67.5%

Step 8;

Output $\approx$68% prediction of HIV diagnosis. This MAVSCOT predicted result reveals the possible severity of HIV in the female patient’s body.

See Table S10 to see the MAVSCOT software predicted results. This results reveal that the female patient has been diagnosed with HIV at the severe stage. The patient seems to have entered into the advanced chronic HIV infection stage [See Table S2].
