# Supplementary material for: MAVSCOT: A fuzzy logic-based HIV diagnostic system with indigenous multi-lingual interfaces for rural Africa
Source: PLoS One. 2020 Nov 6;15(11):e0241864. doi: 10.1371/journal.pone.0241864 (PMC7647102; doi:10.1371/journal.pone.0241864)
Supplement: S1 File — This file consists of all tables, figures, description, and illustration of an extra example on the use of MAVSCOT software. (DOC) [file pone.0241864.s025.doc]

| **Collection of HIV symptomatic Data** | **HIV Symptoms of patients [PLWHIV] obtained from medical and scientific literature** | **References** |
| --- | --- | --- |
| Collection 1 | Poor sleep, Muscle aches/Joint pain, Fatigue, Anxiety/Nervous, Sadness, Numbness/Pain in the feet, Headache, Memory loss, Sex Problems, Cough,/Shortness of breath, Fever/Chills, Sweats, Dizzy/Dizziness, Bloating/Abdominal pain, Poor appetite, Diarrhea, Nausea/Vomiting, Loss of appetite, Rash, Weight Loss | Wilson et. al., 2016 |
| Collection 2 | Fatigue, Fevers, Dizziness,Hand/foot pain, Memory loss, Nausea, Diarrhea, Sadness, Sleep trouble, Skin problems Cough, Headache, Sweats/chills, Weight loss, Appetite loss, Eye trouble, Mouth pain, Mouth infection, Chest pain, Trouble breathing Runny nose, Abdominal pain, Trouble urinating, Muscle/joint pain | Justice et al., 2001 |
| Collection 3 | Symptoms frequently reported by People Living with HIV(PLWHIV):  Abdominal pain, Anxiety, Changes in body weight/fat, Cognitive decline, Diarrhea, Fatigue, Fever or night sweats, Headaches, Insomnia, Joint pain/stiffness, Loss of strength, Muscle pain, Nausea/vomiting ,Peripheral neuropathy, Reduction in appetite, Sadness, Sexual problems, Shortness of breath/cough, Skin problems | Wilson et. al., 2014 |
| Collection 4 | Trouble falling asleep, Weakness, Numbness/tingling, Poor appetite, Nausea/upset stomach, Hot or cold spells, Shortness of breath,  Faintness/dizziness, Pain in heart/chest | Hudson et al., 2003 |
| Collection 5 | Dementia | Ghafouri et al., 2006 |

Table S1: HIV Symptoms of HIV patients obtained from medical and scientific literature

| **Software Predicted Percentage Range** | **Software Degree of HIV intensity** | **Possible HIV Stage in the Medical field** |
| --- | --- | --- |
| 0%-24% | Mild | Acute HIV Infection –sharp drop in concentration of circulating CD4+T cells (Hernandez-Vargas and Middleton, 2013) |
| 25%-49% | Moderate | Chronic HIV Infection(Clinical latency) CD4+T cells circulation – near normal, drop in viral loads (Hernandez-Vargas and Middleton, 2013) |
| 50%-74% | Severe | Chronic HIV infection(Clinical latency)/ (drop in viral loads)/ CD4+T cells circulation – near normal, drop in viral loads (Hernandez-Vargas and Middleton, 2013) |
| 75%-100% | Very Severe | Full-blown AIDS(most severe stage) – CD4 < 200 Cells/mm3 (Hernandez-Vargas and Middleton, 2013) |

Table S2. Interpretation of HIV diagnosed values and range

| **S/N** | **Patient’s ID** | **Gender** | **HIV Symptoms** |
| --- | --- | --- | --- |
| 1 | PID1 | Male | Abnormal swelling  Anxiety  Dementia  Fatigue  Fever  Headache  Sexual dysfunction  Night sweats  Joint Pain (Rheumatism  Muscle aches  Ulcers in the Genitals  Weight loss  12 Symptoms |
| 2 | PID2 | Female |
| 3 | PID3 | Male |
| 4 | PID4 | Female |
| 5 | PID5 | Female |
| 6 | PID6 | Male |
| **7** | **PID7** | **Male** |

Table S3. Sample data from Patients with 10 HIV symptoms

| Patient ID | Abnormal swelling | Anxiety | Dementia | Fatigue | Fever | Headache | Sexual dysfunction | Night sweats | Joint Pain (Rheumatism | Muscle aches | Ulcers in the Genitals | Weight loss |
| --- | --- | --- | --- | --- | --- | --- | --- | --- | --- | --- | --- | --- |
| PID1 | 3 | 3 | 2 | 3 | 1 | 3 | 3 | 1 | 1 | 3 | 1 | 3 |
| PID2 | 2 | 3 | 1 | 2 | 3 | 1 | 2 | 3 | 1 | 2 | 3 | 1 |
| PID3 | 3 | 1 | 1 | 1 | 1 | 1 | 1 | 1 | 1 | 1 | 1 | 1 |
| PID4 | 3 | 2 | 3 | 2 | 3 | 2 | 3 | 2 | 3 | 2 | 1 | 3 |
| PID5 | 3 | 2 | 1 | 3 | 2 | 1 | 3 | 2 | 1 | 3 | 2 | 1 |
| PID6 | 2 | 1 | 2 | 1 | 2 | 1 | 2 | 1 | 2 | 1 | 2 | 1 |
| **PID7** | **3** | **3** | **2** | **3** | **3** | **3** | **3** | **3** | **3** | **3** | **3** | **1** |

Table S4. Weights assigned to patients by doctors who have interacted with the patients concerned.

Table S4 shows a sample of rating of the patients on HIV diagnosis variables. This table shows the weights assigned to patients by doctors who have interacted with the patients concerned.

| Patient ID | Abnormal swelling | Anxiety | Dementia | Fatigue | Fever | Headache | Sexual dysfunction | Night sweats | Joint Pain (Rheumatism | Muscle aches | Ulcers in the Genitals | Weight loss |
| --- | --- | --- | --- | --- | --- | --- | --- | --- | --- | --- | --- | --- |
| PID1 | 0.67 | 0.67 | 0.33 | 0.67 | 0 | 0.67 | 0.67 | 0 | 0 | 0.67 | 0 | 0.67 |
| PID2 | 0.33 | 0.67 | 0 | 0.33 | 0.67 | 0 | 0.33 | 0.67 | 0 | 0.33 | 0.67 | 0 |
| PID3 | 0.67 | 0 | 0 | 0 | 0 | 0 | 0 | 0 | 0 | 0 | 0 | 0 |
| PID4 | 0.67 | 0.33 | 0.67 | 0.33 | 0.67 | 0.33 | 0.67 | 0.33 | 0.67 | 0.33 | 0 | 0.67 |
| PID5 | 0.67 | 0.33 | 0 | 0.67 | 0.33 | 0 | 0.67 | 0.33 | 0 | 0.67 | 0.33 | 0 |
| PID6 | 0.33 | 0 | 0.33 | 0 | 0.33 | 0 | 0.33 | 0 | 0.33 | 0 | 0.33 | 0 |
| **PID7** | **0.67** | **0.67** | **0.33** | **0.67** | **0.67** | **0.67** | **0.67** | **0.67** | **0.67** | **0.67** | **0.67** | **0** |

Table S5. Derived triangular values for HIV symptoms

| **HIV Symptoms for Patient (PID7)** | **Degree of HIV symptom** | Triangular fuzzy numbers of the HIV symptoms |
| --- | --- | --- |
| Abnormal swelling | Severe | 0.67 |
| Anxiety | Severe | 0.67 |
| Dementia | Moderate | 0.33 |
| Fatigue | Severe | 0.67 |
| Fever | Severe | 0.67 |
| Headache | Severe | 0.67 |
| Sexual dysfunction | Severe | 0.67 |
| Night sweats | Severe | 0.67 |
| Joint Pain (Rheumatism) | Severe | 0.67 |
| Muscle aches | Severe | 0.67 |
| Ulcers in the Genitals | Severe | 0.67 |
| Weight loss | Mild | 0 |

Table S6. Values entered for patient 7 (with ID = PID7).

| Rule No. | Abnormal swelling | Anxiety | Dementia | Fatigue | Fever | Headache | Sexual dysfunction | Night sweats | Joint Pain (Rheumatism | Muscle aches | Ulcers in the Genitals | Weight loss | ALL Life Style Questions | Conclusion  HIV presence predicted |
| --- | --- | --- | --- | --- | --- | --- | --- | --- | --- | --- | --- | --- | --- | --- |
| 1 | Mild | Moderate | Severe | Severe | Moderate | Severe | Moderate | Moderate | Severe | Moderate | Moderate | Moderate | Yes | 55.32% HIV Severe |
| 2 | Moderate | Moderate | Mild | Mild | Mild | Mild | Mild | Mild | Mild | Mild | Mild | Mild | Yes | 44.72% HIV Moderate |
| 3 | Mild | Mild | Mild | Mild | Mild | Mild | Mild | Mild | Mild | Mild | Mild | Mild | Yes | 12.44 HIV Mild |
| 4 | Moderate | Mild | Mild | Mild | Mild | Mild | Severe | Severe | Severe | Severe | Severe | Severe | Yes | 46.55% HIV Moderate |
| 5 | Mild | Severe | Mild | Severe | Mild | Severe | Mild | Severe | Mild | Mild | Mild | Mild | Yes | 44.27% HIV Moderate |
| 6 | Mild | Severe | Mild | Severe | Mild | Severe | Severe | Mild | Severe | Mild | Severe | Mild | Yes | 48.57% HIV Moderate |
| 7 | Severe | Mild | Severe | Mild | Severe | Mild | Severe | Mild | Severe | Severe | Severe | Severe | Yes | 50.21  % HIV Severe |
| 8 | Mild | Mild | Mild | Mild | Severe | Mild | Severe | Mild | Severe | Mild | Mild | Mild | Yes | 47.29% HIV Moderate |
| 9 | Moderate | Moderate | Moderate | Moderate | Moderate | Moderate | Moderate | Moderate | Moderate | Moderate | Moderate | Moderate | Yes | 51.54% HIV Severe |
| 10 | Severe | Severe | Moderate | Severe | Severe | Severe | Severe | Severe | Severe | Severe | Severe | Mild | Yes | 57.44% HIV Severe |
| 11 | Mild | Mild | Severe | Severe | Mild | Mild | Severe | Severe | Mild | Mild | Severe | Mild | Yes | 50.51% HIV Severe |
| 12 | Mild | Moderate | Mild | Moderate | Mild | Moderate | Mild | Moderate | Mild | Moderate | Mild | Moderate | Yes | 44.84% HIV Moderate |
| 13 | Mild | Mild | Mild | Mild | Mild | Moderate | Mild | Mild | Moderate | Mild | Mild | Mild | Yes | 42.05% HIV Moderate |
| 14 | Mild | Mild | Mild | Mild | Mild | Mild | Mild | Mild | Mild | Mild | **Moderate** | Mild | Yes | 35.58% HIV Moderate |
| 15 | Mild | Moderate | Severe | Mild | Moderate | Severe | Mild | Moderate | Severe | Mild | Moderate | Severe | Yes | 47.5% HIV Moderate |
| 16 | Severe | Mild | Moderate | Severe | Mild | Moderate | Severe | Mild | Moderate | Severe | Mild | Moderate | Yes | 52.57% HIV Severe |
| 17 | Moderate | Moderate | Moderate | Mild | Mild | Mild | Severe | Severe | Severe | Moderate | Moderate | Moderate | Yes | 54.19% HIV Severe |
| 18 | Mild | Severe | Moderate | Mild | Severe | Moderate | Mild | Severe | Moderate | Mild | Severe | Moderate | Yes | 47.28% HIV Moderate |
| 19 | Moderate | Mild | Mild | Severe | Mild | Mild | Moderate | Mild | Mild | Severe | Mild | Mild | Yes | 47.81% HIV Moderate |
| 20 | Severe | Moderate | Moderate | Severe | Moderate | Moderate | Severe | Moderate | Moderate | Severe | Moderate | Moderate | Yes | 55.75% HIV Severe |
| 21 | Mild | Severe | Severe | Moderate | Severe | Severe | Mild | Severe | Severe | Moderate | Severe | Severe | Yes | 52.72% HIV Severe |

Table S7. Fuzzy Rule Base for HIV – using 21 rules

From Table S7, some interpretations were provided for some of the rules (Rules, 1, 10, and 21) as follows:

**Rule 1:** IF Abnormal Swelling = Mild and Anxiety = Moderate, and Dementia = Severe, and Fatigue = Severe, and Fever = Moderate and Headache = Severe and Sexual Dysfunction = Moderate and Night Sweats = Moderate and Joint Pain = Severe and Muscle Aches = Moderate and Ulcers in the Genitals = Moderate and Weight Loss = Moderate and Patient has multiple sex partners, and Patient has shared unsterilized objects with others and Patient has had unprotected sex, and Patient has undergone unscreened blood transfusion and Patient is aware of HIV/AIDS and Patient has been self-administering sexual stimulants THEN the possible presence of HIV in the patients body = **SEVERE**

**Rule 10:** IF Abnormal Swelling = Severe and Anxiety = Severe, and Dementia = Severe, and Fatigue = Severe, and Fever = Severe and Headache = Severe and Sexual Dysfunction = Severe and Night Sweats = Severe and Joint Pain = Severe and Muscle Aches = Severe and Ulcers in the Genitals = Severe and Weight Loss = Severe and Patient has multiple sex partners, and Patient has shared unsterilized objects with others and Patient has had unprotected sex, and Patient has undergone unscreened blood transfusion and Patient is aware of HIV/AIDS and Patient has been self-administering sexual stimulants THEN the possible presence of HIV in the patients body = **SEVERE**

**Rule 21:** IF Abnormal Swelling = Mild and Anxiety = Severe, and Dementia = Severe, and Fatigue = Severe, and Fever = Moderate and Headache = Severe and Sexual Dysfunction = Moderate and Night Sweats = Moderate and Joint Pain = Severe and Muscle Aches = Moderate and Ulcers in the Genitals = Moderate and Weight Loss = Moderate and Patient has multiple sex partners, and Patient has shared unsterilized objects with others and Patient has had unprotected sex, and Patient has undergone unscreened blood transfusion and Patient is aware of HIV/AIDS and Patient has been self-administering sexual stimulants THEN the possible presence of HIV in the patients body = **SEVERE**

| Rule No. | Abnormal swelling | Anxiety | Dementia | Fatigue | Fever | Headache | Sexual dysfunction | Night sweats | Joint Pain (Rheumatism | Muscle aches | Ulcers in the Genitals | Weight loss | Non-Zero Mimimum Values |
| --- | --- | --- | --- | --- | --- | --- | --- | --- | --- | --- | --- | --- | --- |
| 1 | - | - | - | 0.67 | - | 0.67 | - | - | 0.67 | - | - | - | 0.67 |
| 2 | - | - | - | - | - | - | - | - | - | - | - | 0 |  |
| 3 | - | - | - | - | - | - | - | - | - | - | - | 0 |  |
| 4 | - | - | - | - | - | - | 0.67 | 0.67 | 0.67 | 0.67 | 0.67 | - | 0.67 |
| 5 | - | 0.67 | - | 0.67 | - | 0.67 | - | 0.67 | - | - | - | 0 | 0.67 |
| 6 | - | 0.67 | - | 0.67 | - | 0.67 | 0.67 | - | 0.67 | - | 0.67 | 0 | 0.67 |
| 7 | 0.67 | - | - | - | 0.67 | - | 0.67 | - | 0.67 | 0.67 | 0.67 | - | 0.67 |
| 8 | - | - | - | - | 0.67 | - | 0.67 | - | 0.67 | - | - | 0 | 0.67 |
| 9 | - | - | 0.33 | - | - | - | - | - | - | - | - | - | 0.33 |
| 10 | 0.67 | 0.67 | - | 0.67 | 0.67 | 0.67 | 0.67 | 0.67 | 0.67 | 0.67 | 0.67 | - | 0.67 |
| 11 | - | - | 0.67 | 0.67 | - | - | 0.67 | 0.67 | - | - | 0.67 | 0 |  |
| 12 | - | - | - | - | - | - | - | - | - | - | - | - |  |
| 13 | - | - | - | - | - | - | - | - | - | - | - | 0 |  |
| 14 | - | - | - | - | - | - | - | - | - | - | - | 0 |  |
| 15 | - | - | - | - | - | - | - | - | - | - | - | - |  |
| 16 | 0.67 | - | 0.33 | 0.67 | - | - | 0.67 | - | - | 0.67 | - | - | 0.33 |
| 17 | - | - | 0.33 | - | - | - | 0.67 | 0.67 | 0.67 | - | - | - | 0.33 |
| 18 | - | 0.67 | 0.33 | - | 0.67 | - | - | 0.67 | - | - | - | - | 0.33 |
| 19 | - | - | - | 0.67 | - | - | - | - | - | 0.67 | - | 0 | 0.67 |
| 20 | 0.67 | - | 0.33 | 0.67 | - | - | 0.67 | - | - | 0.67 | - | - | 0.33 |
| 21 | - | 0.67 | 0.67 | - | 0.67 | 0.67 | - | 0.67 | 0.67 | - | 0.67 | - | 0.67 |

Table S8. Rule-Based evaluation for Patient 7(PID7), based on the Rule base specified in Table S7 Fuzzy Rule Base for HIV – using 21 rules

You map Table S6 unto Table S7 to obtain Table S8 (non-zero minimum values)

| Rule No. | Abnormal swelling | Anxiety | Dementia | Fatigue | Fever | Headache | Sexual dysfunction | Night sweats | Joint Pain (Rheumatism | Muscle aches | Ulcers in the Genitals | Weight loss | Non-Zero Mimimum Values and HIV severity |
| --- | --- | --- | --- | --- | --- | --- | --- | --- | --- | --- | --- | --- | --- |
| 1 | - | - | - | 0.67 | - | 0.67 | - | - | 0.67 | - | - | - | 0.67 Severe |
| 2 | - | - | - | - | - | - | - | - | - | - | - | 0 | Moderate |
| 3 | - | - | - | - | - | - | - | - | - | - | - | 0 | Mild |
| 4 | - | - | - | - | - | - | 0.67 | 0.67 | 0.67 | 0.67 | 0.67 | - | 0.67 Severe |
| 5 | - | 0.67 | - | 0.67 | - | 0.67 | - | 0.67 | - | - | - | 0 | 0.67 Severe |
| 6 | - | 0.67 | - | 0.67 | - | 0.67 | 0.67 | - | 0.67 | - | 0.67 | 0 | 0.67 Severe |
| 7 | 0.67 | - | - | - | 0.67 | - | 0.67 | - | 0.67 | 0.67 | 0.67 | - | 0.67 Severe |
| 8 | - | - | - | - | 0.67 | - | 0.67 | - | 0.67 | - | - | 0 | 0.67 Severe |
| 9 | - | - | 0.33 | - | - | - | - | - | - | - | - | - | 0.33 Mild |
| 10 | 0.67 | 0.67 | - | 0.67 | 0.67 | 0.67 | 0.67 | 0.67 | 0.67 | 0.67 | 0.67 | - | 0.67 Severe |
| 11 | - | - | 0.67 | 0.67 | - | - | 0.67 | 0.67 | - | - | 0.67 | 0 | 0.67 Severe |
| 12 | - | - | - | - | - | - | - | - | - | - | - | - | - Moderate |
| 13 | - | - | - | - | - | - | - | - | - | - | - | 0 | Mild |
| 14 | - | - | - | - | - | - | - | - | - | - | - | 0 | Mild |
| 15 | - | - | - | - | - | - | - | - | - | - | - | - | - Moderate |
| 16 | 0.67 | - | 0.33 | 0.67 | - | - | 0.67 | - | - | 0.67 | - | - | 0.33 Moderate |
| 17 | - | - | 0.33 | - | - | - | 0.67 | 0.67 | 0.67 | - | - | - | 0.33 Moderate |
| 18 | - | 0.67 | 0.33 | - | 0.67 | - | - | 0.67 | - | - | - | - | 0.33 Moderate |
| 19 | - | - | - | 0.67 | - | - | - | - | - | 0.67 | - | 0 | 0.67 Severe |
| 20 | 0.67 | - | 0.33 | 0.67 | - | - | 0.67 | - | - | 0.67 | - | - | 0.33 Moderate |
| 21 | - | 0.67 | 0.67 | - | 0.67 | 0.67 | - | 0.67 | 0.67 | - | 0.67 | - | 0.67 Severe |

Table S9. List of Rules that produced non-zero minimum values. These sets of rules are: Rules 1,4,5,6, 7, 8, 9, 10, 11, 16, 17, 18, 19, 20, 21. These can be respectively classified as follows:

Mild = None

Moderate=R4, R5, R6, R8, R18,

Severe =R1, R7, R9, R10, R11, R16, R17, R19, R20, R21,

| **HIV predicted results of the English Version of the Multilingual indigenous Informatics(MAVSCOT) Software** | **HIV predicted results of the Afrikaans Version of the Multilingual indigenous Informatics(MAVSCOT) Software** | **HIV predicted results of the IsiXhosa Version of the Multilingual indigenous Informatics(MAVSCOT) Software** | **HIV predicted results of the Zulu Version of the Multilingual indigenous Informatics(MAVSCOT) Software** |
| --- | --- | --- | --- |
| 68.04%68% HIV presence | 68.04%68% HIV presence | 68.04%68% HIV presence | 68.24%68% HIV presence (possible HIV presence or viral load of HIV within the human body) |

Table S10: Predicted results of MAVSCOT from the second example of a female HIV patient

| References | Pazzani et al., 1997) | Tucker et al., 2013). | *Ebrahimi et al., 2013) | *Atalay et al., 1999), | Oluwagbemi et al., ) MAVSCOT |
| --- | --- | --- | --- | --- | --- |
| Comparative Features/Factors |
| 1. Description | Pazzani and colleagues, applied the knowledge of rule-based expert systems to the management of HIV-infected patients. Their system encodes information from existing literature of known drug resistant mutations. | Tucker and colleagues examined how a telephone-based IVR (Interactive Voice Response) self-monitoring system can be used to access daily HIV anti-retroviral medication adherence | Ebrahimi and colleagues developed an intelligent AIDS/HIV web-based medical consulting system which provides consulting services on systematic textual data  *Web-based application | Atalay and colleagues developed an interactive web-based HIV patient care expert systems | Multilingual Indigenous HIV Informatics Software for South Africa  *Standalone application |
| 2. Multilingual Features (Text-based) | Nil | Nil | Nil | Nil | Yes  Four(4) languages – English language and three(3) South African indigenous languages-Afrikaans, IsiXhosa and Zulu |
| 3.Voice-Enbled/Speech-based features | Nil | Yes  An Interactive Voice Response (IVR) system was developed. | Nil | Nil  Although interactive | Yes  *Not interactive but system. *Pronunciation and Intonations in English language was good.  *Although the pronunciations and intonations of speech-features of Afrikaans, IsiXhosa and Zulu are not perfect. More work still need to be done. |
| 4. HIV Predictive Feature , Advisory Features (Functionalities) | Yes  *applied the knowledge of rule-based expert systems.  * System encodes information from existing literature of known HIV drug resistant mutations. | Nil | Nil | Nil  *it’s a web-based, question-answer session, patient-care system, based on inference engine and knowledge base | Yes  Fuzzy-logic-rule-based predictive mechanism  *it has inference engine for the fuzzy concepts and knowledge base to store rules and HIV symptoms |
| 5. Input Data | *information from the literature of known HIV drug resistance mutations | *Voice in English only | Textual data | Textual data | *HIV (textual data) symptoms to be selected  *Demographic textual data selection  *History of Health record data selection |
| 6. Output data | Textual data | *Voice in English only | *Textual data  *Provides answers to inquiries made online about HIV | Textual data | *Predicts HIV intensity (%) in numbers and in Voice/speech  *Prescribes HIV drugs with the corresponding dosage (text and voice/speech)  *Provides advice to patients(text and voice/speech)  *Provides recommendation to patients.(text and voice/speech) |

Table S11: Comparison of existing HIV voice-enabled expert system/software with HIV multilingual indigenous informatics software

|  | Symptoms |  |  |  |  |  |  |  |  |  |  |  |  |  |  |  |  |  |  |  |  |  |  |  | Q1 | Q2 | Q3 | Q4 | Q5 | Q6 | Conclusio[Prediction] |  |
| --- | --- | --- | --- | --- | --- | --- | --- | --- | --- | --- | --- | --- | --- | --- | --- | --- | --- | --- | --- | --- | --- | --- | --- | --- | --- | --- | --- | --- | --- | --- | --- | --- |
| Rule Number | Weight Loss  [S39] | Vomitting[S38] | Ulcer on the Genitals[S36] | Swollen Lymph Nodes[S35] | Stomach Upset[S34] | Soreness of the Vagina[S33] | Sexual Dysfunction[S30] | Painful Urination[S28] | Painful Intercourse[S27 | Pain U.R. Abdomen[S26 | Missed periods[S21] | Lower Abdominal Pain[S19] | Joint Pain[S18 | Itching in the Vaginal Area[S17 | Heavier or Lighter Periods[S16 | Gonorrhoea[S14] | Forgetfulness[S13] | Depression[S9 | Diarrhoea[S10 | Dementia(Memory Loss  [S8 | Body Temperature[S5] | Anxiety[S3] | Abnormal vaginal discharge[S2] | Abdominal Swelling[S1] |  |  |  |  |  |  |  |  |
| 1 | Mild | Moderate | Severe | Severe | Moderate | Severe | Moderate | Moderate | Moderate | Mild | Moderate | Severe | Severe | Moderate | Severe | Moderate | Moderate | Moderate | Mild | Moderate | Severe | Severe | Moderate | Severe | Y | Y | Y | Y | N | Y | English[62.19%];  Afrikaans[62.19%]  IsiXhosa[62.19%]  Zulu [62.19%] | SEVERE |
| 2 | Moderate | Moderate | Mild | Mild | Mild | Mild | Mild | Mild | Mild | Moderate | Moderate | Mild | Mild | Mild | Mild | Mild | Mild | Mild | Moderate | Moderate | Mild | Mild | Mild | Mild | N | Y | Y | Y | N | Y | English  [45.91%]  Afrikaans[45.91%  IsiXhosa[45.91%  Zulu [45.91% | MODERATE |
| 3 | Mild | Mild | Mild | Mild | Mild | Mild | Mild | Mild | Mild | Mild | Mild | Mild | Mild | Mild | Mild | Mild | Mild | Mild | Mild | Mild | Mild | Mild | Mild | Mild | Y | Y | Y | Y | Y | N | English  [17.69%]  Afrikaans[17.69%]  IsiXhosa[[17.69%]  Zulu [17.69%] | MILD |
| 4 | Moderate | Mild | Mild | Mild | Mild | Mild | Severe | Severe | Severe | Moderate | Mild | Mild | Mild | Mild | Mild | Severe | Severe | Severe | Moderate | Mild | Mild | Mild | Mild | Mild | N | N | Y | Y | Y | N | English  [52.17%]  Afrikaans[52.17%]  IsiXhosa[[52.17%]  Zulu [52.17%] | SEVERE |
| 5 | Mild | Mild | Mild | Mild | Severe | Moderate | Severe | Mild | Mild | Mild | Mild | Mild | Mild | Severe | Moderate | Severe | Mild | Mild | Mild | Mild | Mild | Mild | Severe | Moderate | N | N | N | N | N | Y | English  [47.31%]  Afrikaans[47.31%]  IsiXhosa[[47.31%]  Zulu [47.31%] | MODERATE |
| 6 | Mild | Severe | Mild | Severe | Mild | Severe | Mild | Mild | Mild | Mild | Severe | Mild | Severe | Mild | Severe | Mild | Mild | Mild | Mild | Severe | Mild | Severe | Mild | Severe | Y | N | Y | N | Y | N | English  [53.42%]  Afrikaans[53.42%]  IsiXhosa[[53.42%]  Zulu [53.42%] | SEVERE |
| 7 | Severe | Mild | Severe | Mild | Severe | Mild | Severe | Mild | Severe | Severe | Mild | Severe | Mild | Severe | Mild | Severe | Mild | Severe | Severe | Mild | Severe | Mild | Severe | Mild | N | Y | N | Y | N | Y | English  [57.52%]  Afrikaans[57.52%]  IsiXhosa[[57.52%]  Zulu [57.52%] | SEVERE |
| 8 | Mild | Mild | Mild | Mild | Severe | Mild | Severe | Mild | Mild | Mild | Mild | Mild | Mild | Severe | Mild | Severe | Mild | Mild | Mild | Mild | Mild | Mild | Severe | Mild | N | N | N | Y | Y | Y | English  [46.15%]  Afrikaans[46.15%]  IsiXhosa[[46.15%]  Zulu [46.15%] | MODERATE |
| 9 | Moderate | Moderate | Moderate | Moderate | Moderate | Moderate | Moderate | Moderate | Moderate | Moderate | Moderate | Moderate | Moderate | Moderate | Moderate | Moderate | Moderate | Moderate | Moderate | Moderate | Moderate | Moderate | Moderate | Moderate | N | N | N | N | Y | Y | English  [56.44%]  Afrikaans[56.44%]  IsiXhosa[[56.44%]  Zulu [56.44%] | SEVERE |
| 10 | Severe | Severe | Severe | Severe | Severe | Severe | Severe | Severe | Severe | Severe | Severe | Severe | Severe | Severe | Severe | Severe | Severe | Severe | Severe | Severe | Severe | Severe | Severe | Severe | Y | Y | Y | Y | Y | Y | English  [73.08%]  Afrikaans[73.08%]  IsiXhosa[[73.08%]  Zulu [73.08%] | SEVERE |
| 11 | Mild | Mild | Severe | Severe | Mild | Mild | Severe | Severe | Mild | Mild | Severe | Severe | Mild | Mild | Severe | Severe | Mild | Mild | Severe | Severe | Mild | Mild | Severe | Severe | N | N | N | N | N | N | English  [57.24%]  Afrikaans[57.24%]  IsiXhosa[[57.24%]  Zulu [57.24%] | SEVERE |
| 12 | Severe | Severe | Severe | Severe | Severe | Severe | Severe | Severe | Severe | Severe | Severe | Severe | Severe | Severe | Severe | Severe | Severe | Severe | Severe | Severe | Severe | Severe | Severe | Severe | N | N | N | N | N | N | English  [72.64%]  Afrikaans[72.64%]  IsiXhosa[[72.64%]Zulu [72.64%] | SEVERE |
| 13 | Mild | Mild | Mild | Moderate | Moderate | Moderate | Moderate | Moderate | Severe | Mild | Mild | Mild | Moderate | Moderate | Moderate | Moderate | Moderate | Severe | Mild | Mild | Mild | Moderate | Moderate | Moderate | Y | Y | N | N | Y | Y | English  [53.55%]  Afrikaans[53.55%]  IsiXhosa[[53.55%]Zulu [53.55%] | SEVERE |
| 14 | Severe | Severe | Severe | Severe | Severe | Moderate | Moderate | Severe | Severe | Severe | Severe | Severe | Severe | Severe | Moderate | Moderate | Severe | Severe | Severe | Severe | Severe | Severe | Severe | Moderate | N | N | N | Y | N | Y | English  [71.59%]  Afrikaans[71.59%]  IsiXhosa[[71.59%]Zulu [71.59%] | SEVERE |

Table S12: Fuzzy Rule Base for the HIV Multilingual Informatics Software in English and three (3) indigenous South African languages – Using 14 Rules

| **HIV Symptoms Code** | **HIV Symptoms of HIV patients obtained from Medical and Scientific literature** | **Severity of HIV infections** | **Rating on Variables** | **Triangular Fuzzification Function values** |
| --- | --- | --- | --- | --- |
| S39 | Weight Loss | Severe | 3 | ((3-1)/3) = 0.67 |
| S38 | Vomiting | Severe | 3 | ((3-1)/3) = 0.67 |
| S36 | Ulcer at Genitals | Severe | 3 | ((3-1)/3) = 0.67 |
| S35 | Swollen Lymph Nodes | Severe | 3 | ((3-1)/3) = 0.67 |
| S34 | Stomach Upset | Severe | 3 | ((3-1)/3) = 0.67 |
| S33 | Soreness of the Vagina | Moderate | 2 | ((3-2)/3) = 0.33 |
| S30 | Sexual Dysfunction | Severe | 3 | ((3-1)/3) = 0.67 |
| S28 | Painful Urination | Severe | 3 | ((3-1)/3) = 0.67 |
| S27 | Painful Intercourse | Severe | 3 | ((3-1)/3) = 0.67 |
| S26 | Pain in the Upper right abdomen | Severe | 3 | ((3-1)/3) = 0.67 |
| S21 | Missed Periods | Severe | 3 | ((3-1)/3) = 0.67 |
| S19 | Lower Abdominal pain | Mild | 1 | ((1-1)/3) = 0 |
| S18 | Joint Pain (Rheumatism) | Severe | 3 | ((3-1)/3) = 0.67 |
| S17 | Itching in the Vagina area | Severe | 3 | ((3-1)/3) = 0.67 |
| S16 | Heavier or Lighter Periods | Severe | 3 | ((3-1)/3) = 0.67 |
| S14 | Gonorrhoea | Severe | 3 | ((3-1)/3) = 0.67 |
| S13 | Forgetfulness | Moderate | 2 | ((3-2)/3) = 0.33 |
| S9 | Depression | Severe | 3 | ((3-1)/3) = 0.67 |
| S10 | Diarrhoea | Severe | 3 | ((3-1)/3) = 0.67 |
| S8 | Dementia (MemoryLoss) | Severe | 3 | ((3-1)/3) = 0.67 |
| S5 | Body Temperature | Severe | 3 | ((3-1)/3) = 0.67 |
| S3 | Anxiety | Mild | 1 | ((1-1)/3) = 0 |
| S2 | Abnormal Vagina discharge | Severe | 3 | ((3-1)/3) = 0.67 |
| S1 | Abdominal Swelling | Severe | 3 | ((3-1)/3) = 0.67 |

**Table S13: Symptoms, Severity, Rating on Variables of 24 HIV Symptoms of Patients from Medical and Scientific Literature**

| **Symptoms Codes** | S39 | S38 | S36 | S35 | S34 | S33 | S30 | S28 | S27 | S26 | S21 | S19 | S18 | S17 | S16 | S14 | S13 | S9 | S10 | S8 | S5 | S3 | S2 | S1 |
| --- | --- | --- | --- | --- | --- | --- | --- | --- | --- | --- | --- | --- | --- | --- | --- | --- | --- | --- | --- | --- | --- | --- | --- | --- |
| **Triangular Fuzzy Function**  **Values** | 0.67 | 0.67 | 0.67 | 0.67 | 0.67 | 0.33 | 0.67 | 0.67 | 0.67 | 0.67 | 0.67 | 0 | 0.67 | 0.67 | 0.67 | 0.67 | 0.33 | 0.67 | 0.67 | 0.67 | 0.67 | 0 | 0.67 | 0.67 |

**Table S14:** Table showing the generation of Triangular Fuzzy Function Values

|  | Symptoms |  |  |  |  |  |  |  |  |  |  |  |  |  |  |  |  |  |  |  |  |  |  |  | Conclusio[Prediction] | Non Zero Minimum Value |
| --- | --- | --- | --- | --- | --- | --- | --- | --- | --- | --- | --- | --- | --- | --- | --- | --- | --- | --- | --- | --- | --- | --- | --- | --- | --- | --- |
| Rule Number (R ) | Weight Loss  [S39] | Vomitting[S38] | Ulcer on the Genitals[S36] | Swollen Lymph Nodes[S35] | Stomach Upset[S34] | Soreness of the Vagina[S33] | Sexual Dysfunction[S30] | Painful Urination[S28] | Painful Intercourse[S27 | Pain U.R. Abdomen[S26 | Missed periods[S21] | Lower Abdominal Pain[S19] | Joint Pain[S18 | Itching in the Vaginal Area[S17 | Heavier or Lighter Periods[S16 | Gonorrhoea[S14] | Forgetfulness[S13] | Depression[S9 | Diarrhoea[S10 | Dementia(Memory Loss  [S8 | Body Temperature[S5] | Anxiety[S3] | Abnormal vaginal discharge[S2] | Abdominal Swelling[S1] |  |  |
| 1 | - | 0.67 | 0.67 | - | - | - | - | - | - | - | - | - | 0.67 | - | 0.67 | - | 0.33 | - | - | - | 0.67 | - | - | 0.67 | Moderate | 0.33 |
| 2 | - | - | - | - | - | - | - | - | - | - | - | 0 | - | - | - | - | - | - | - | - | - | 0 | - | - | Mild | 0 |
| 3 | - | - | - | - | - | - | - | - | - | - | - | 0 | - | - | - | - | - | - | - | - | - | 0 | - | - | Mild | 0 |
| 4 | - | - | - | - | - | - | 0.67 | 0.67 | 0.67 | - | - | - | - | - | - | 0.67 | - | 0.67 | - | - | - | - | - | - | Severe | 0.67 |
| 5 | - | - | - | - | 0.67 | 0.33 | 0.67 | - | - | - | - | 0 | - | 0.67 | - | 0.67 | - | - |  | - | - | 0 | 0.67 | - | Moderate | 0.33 |
| 6 |  | 0.67 |  | 0.67 |  |  |  | 0.67 |  | 0.67 |  | 0 |  | 0.67 |  | 0.67 |  | 0.67 |  | 0.67 | - | - | - | 0.67 | Severe | 0.67 |
| 7 | 0.67 | - | 0.67 | - | 0.67 | - | 0.67 | - | 0.67 | 0.67 | - | - | - | 0.67 | - | 0.67 | - | 0.67 | 0.67 | - | 0.67 | 0 | 0.67 | - | Severe | 0.67 |
| 8 | - | - | - | - | 0.67 | - | - | - | - | - | 0 | - | 0.67 | - | 0.67 | - | - | - | - | - | - | 0 | 0.67 | - | Severe | 0.67 |
| 9 | - | - | - | - | - | 0.33 | - | - | - | - | - | - | - | - | - | - | 0.33 | - | - | - | - | - | - | - | Moderate | 0.33 |
| 10 | 0.67 | 0.67 | 0.67 | 0.67 | 0.67 | - | 0.67 | 0.67 | 0.67 | 0.67 | 0.67 | - | 0.67 | 0.67 | 0.67 | 0.67 | - | 0.67 | 0.67 | 0.67 | 0.67 | - | 0.67 | 0.67 | Severe | 0.67 |
| 11 | - | - | 0.67 | 0.67 | - | - | 0.67 | 0.67 | - | - | 0.67 | - | - | - | 0.67 | 0.67 | - | - | 0.67 | 0.67 | - | 0 | 0.67 | 0.67 | Severe | 0.67 |
| 12 | 0.67 | 0.67 | 0.67 | 0.67 | 0.67 | - | 0.67 | 0.67 | 0.67 | 0.67 | 0.67 | - | 0.67 | 0.67 | 0.67 | 0.67 | - | 0.67 | 0.67 | 0.67 | 0.67 | - | 0.67 | 0.67 | Severe | 0.67 |
| 13 | - | - | - | - | - | 0.33 | - | - | 0.67 | - | - | 0 | - | - | - | - | 0.33 | 0.67 | - | - | - | - | - | - | Moderate | 0.33 |
| 14 | 0.67 | 0.67 | 0.67 | 0.67 | 0.67 | 0.33 | - | 0.67 | 0.67 | 0.67 | - | 0.67 | 0.67 | 0.67 | - | - | - | 0.67 | 0.67 | 0.67 | 0.67 | - | 0.67 | - | Moderate | 0.33 |

**Table S15:** Shows the table that generated the Non-zero minimum values


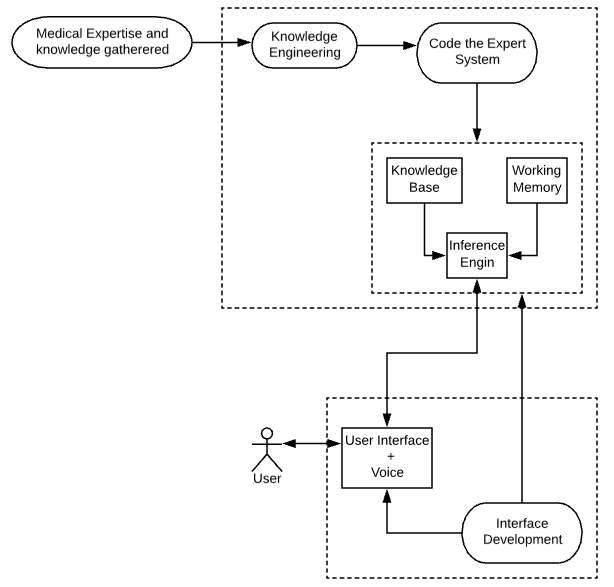


Figure S1: Schematic depiction of the architecture of the Multi-lingual Indigenous HIV Informatics System (MAVSCOT)


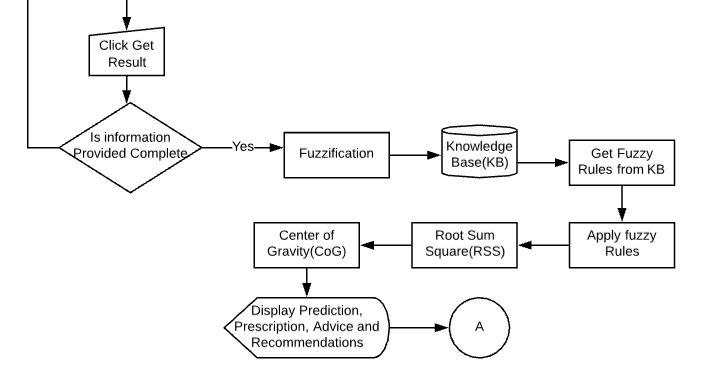

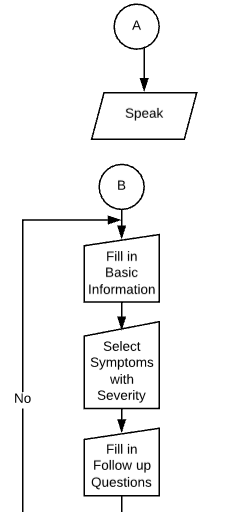

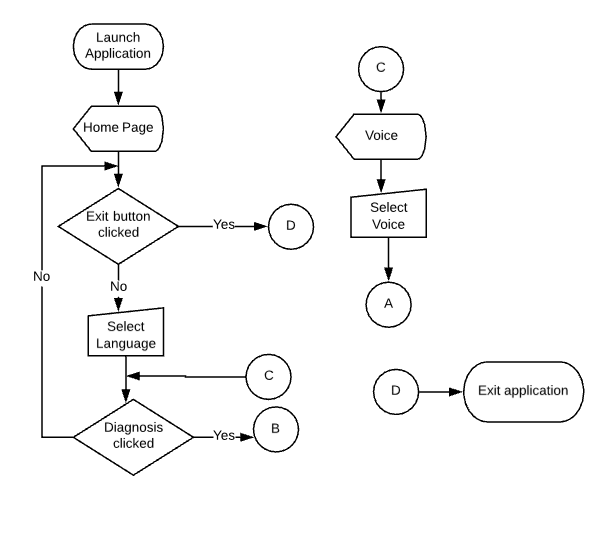


Figure S2: Flowchart of the Multi-lingual Indigenous HIV Informatics System (MAVSCOT)


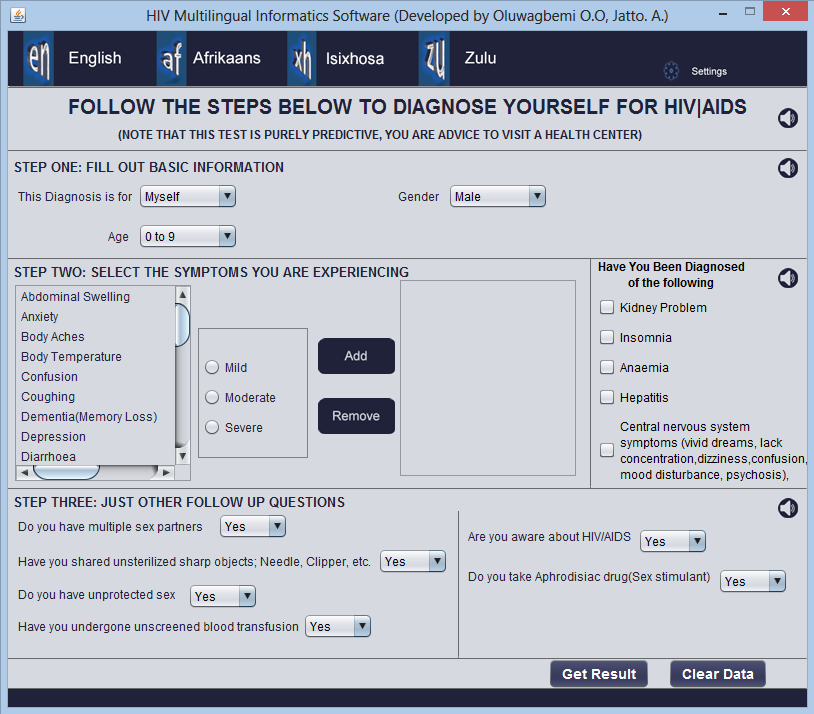


Select your preferred

Language by clicking

Enable or disable audio speaker

Select and add symptoms from the list by just clicking and checking

Select the drop down by simply clicking

Click the button to clear selected symptoms

Click the button to get result

**1**

Figure S3: Graphical User Interface of the MAVSCOT Software.


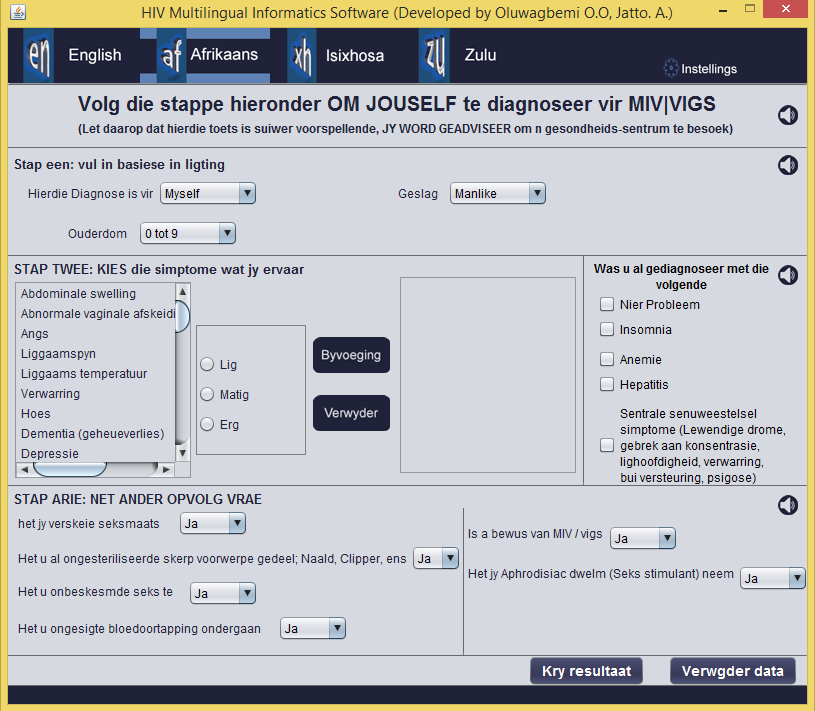


Figure S4. The Graphical User Interface of the HIV Informatics Multi-lingual Voice Enabled Software for Afrikaans language


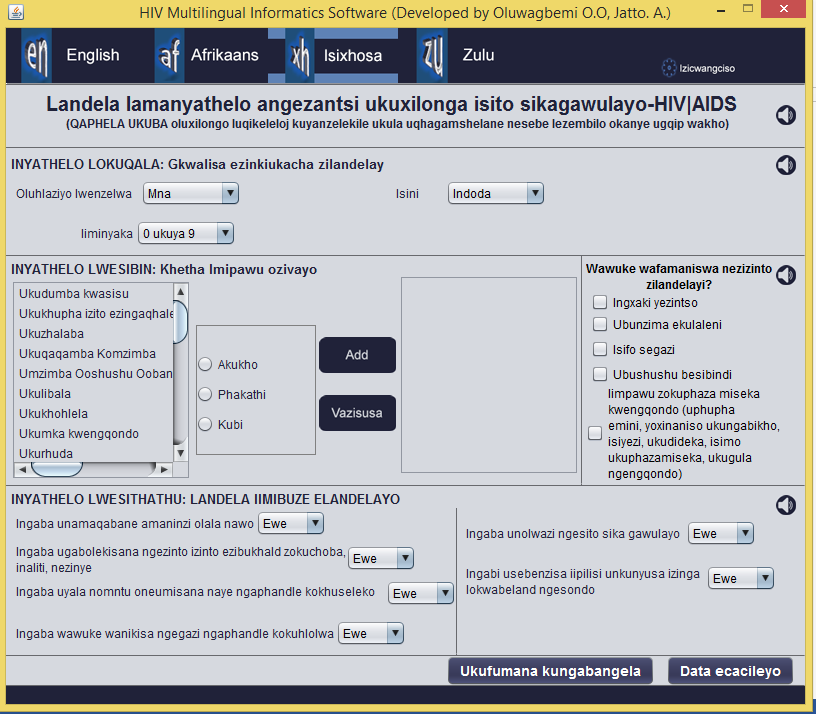
1

Figure S5. The Graphical User Interface of the HIV Informatics Multi-lingual Voice Enabled Software for IsiXhosa language

**
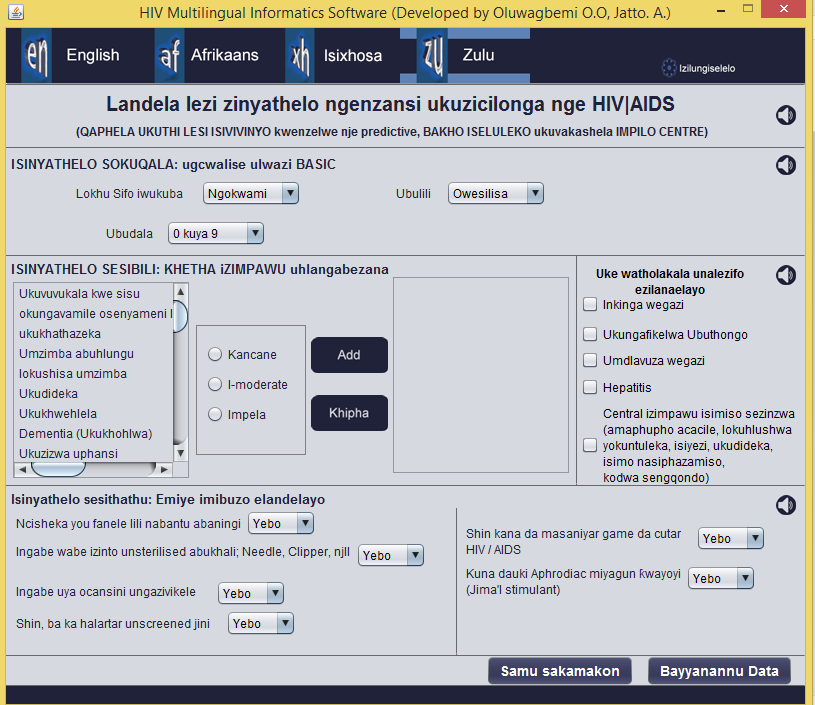
**

1

Figure S6. The Graphical User Interface of the HIV Informatics Multi-lingual Voice Enabled Software for IsiXhosa language


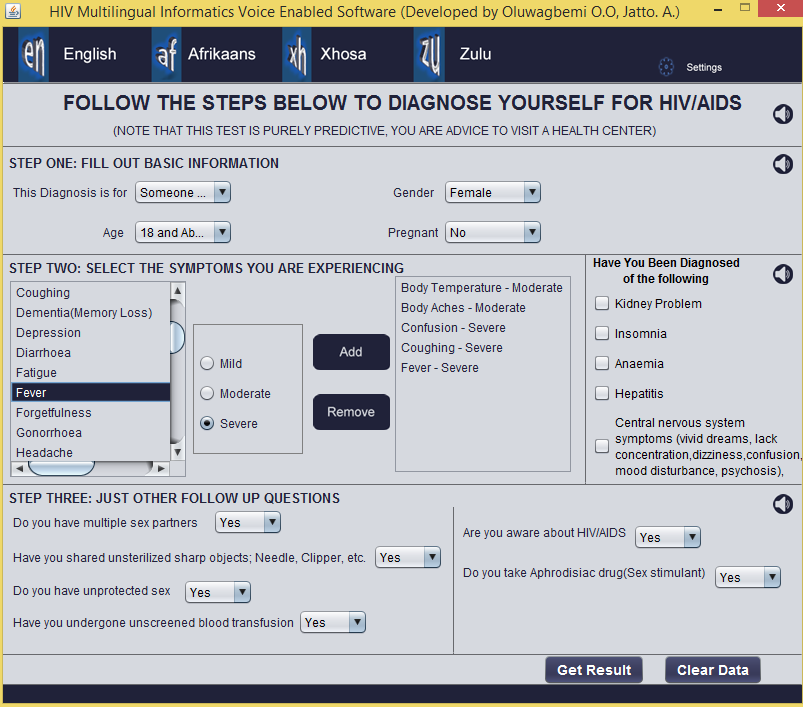


Figure S7. The Graphical User Interface of the HIV Informatics Multi-lingual Voice Enabled Software for English language showing HIV Symptoms keyed-in in English Language

**
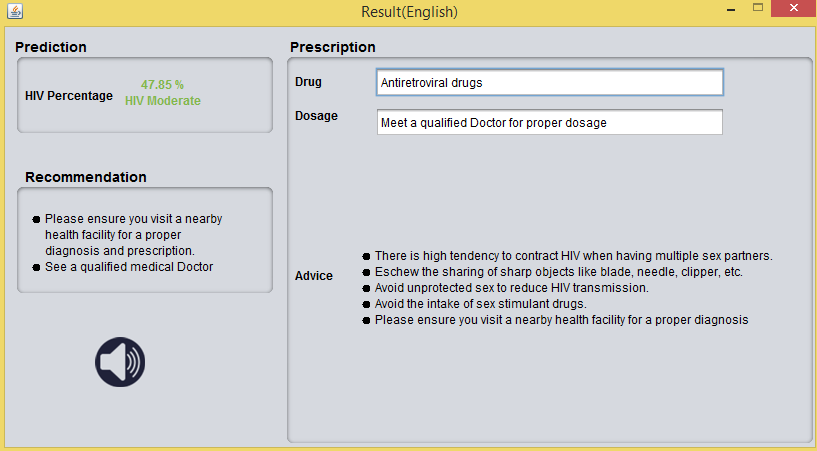
**

Figure S8. The Graphical User Interface of the HIV Informatics Multi-lingual Voice Enabled Software for English language showing HIV diagnosis results


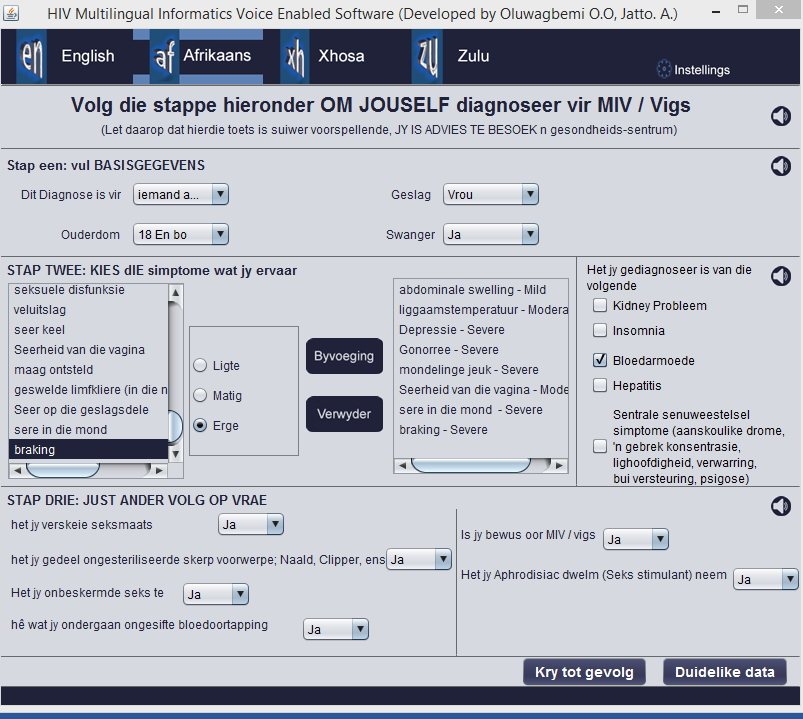


Figure S9. The Graphical User Interface of the HIV Informatics Multi-lingual Voice Enabled Software showing HIV symptoms keyed-in in Afrikaans language.


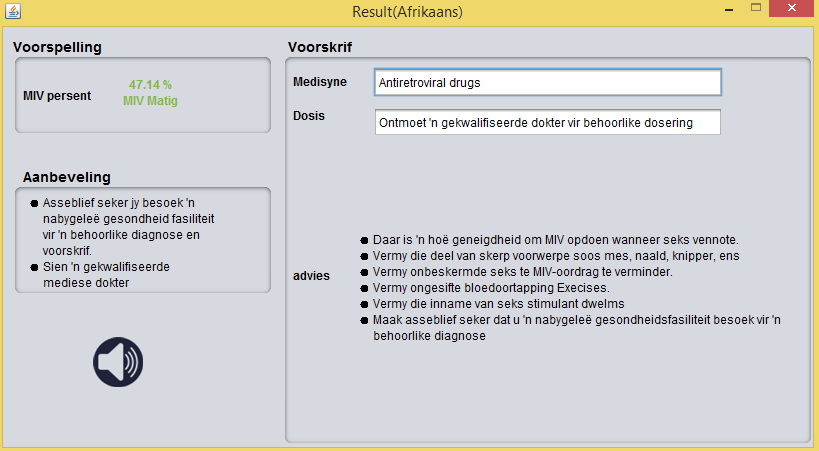


Figure S10. The Graphical User Interface of the HIV Informatics Multi-lingual Voice Enabled Software showing Predicted Results in Afrikaans Language, for selected HIV symptoms


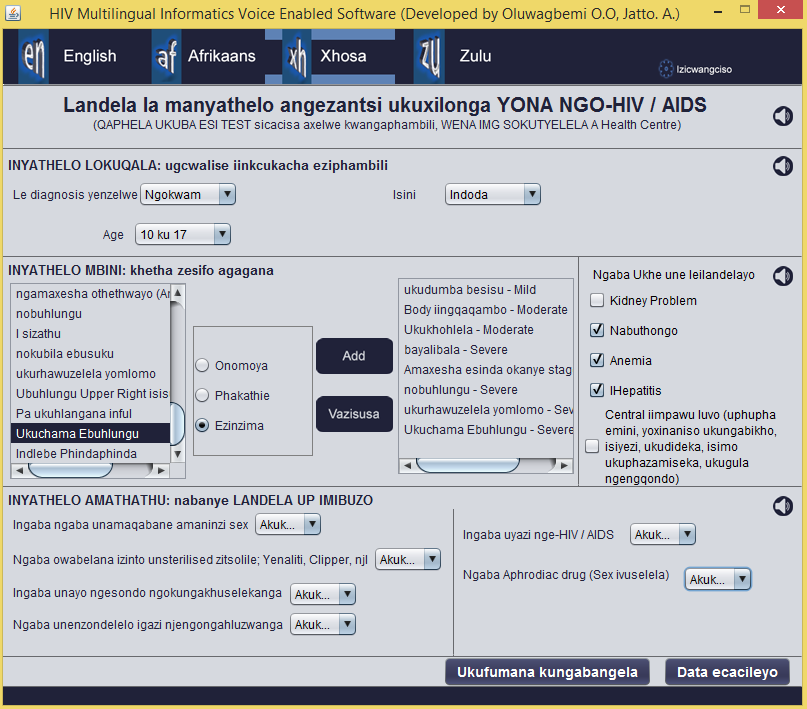
HIV Symptoms keyed-in in Xhosa Language

Figure S11. The Graphical User Interface of the HIV Informatics Multi-lingual Voice Enabled Software showing HIV symptoms keyed-in in Xhosa language.


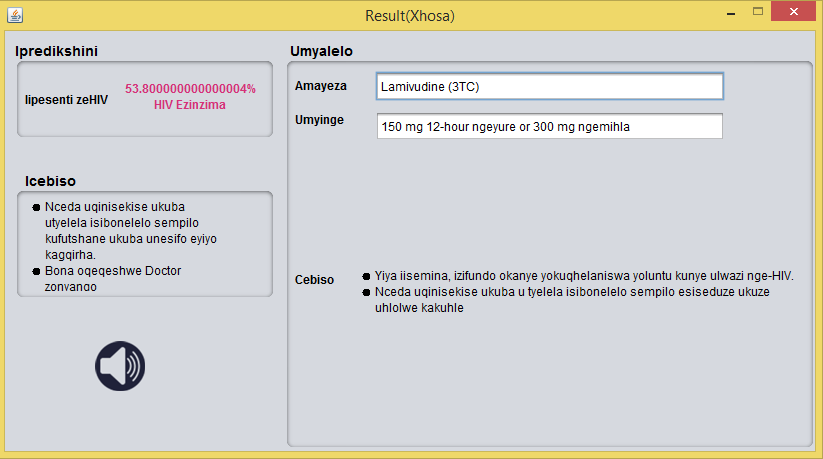


Figure S12. The Graphical User Interface of the HIV Informatics Multi-lingual Voice Enabled Software showing Predicted Results in Xhosa Language, for selected HIV symptoms


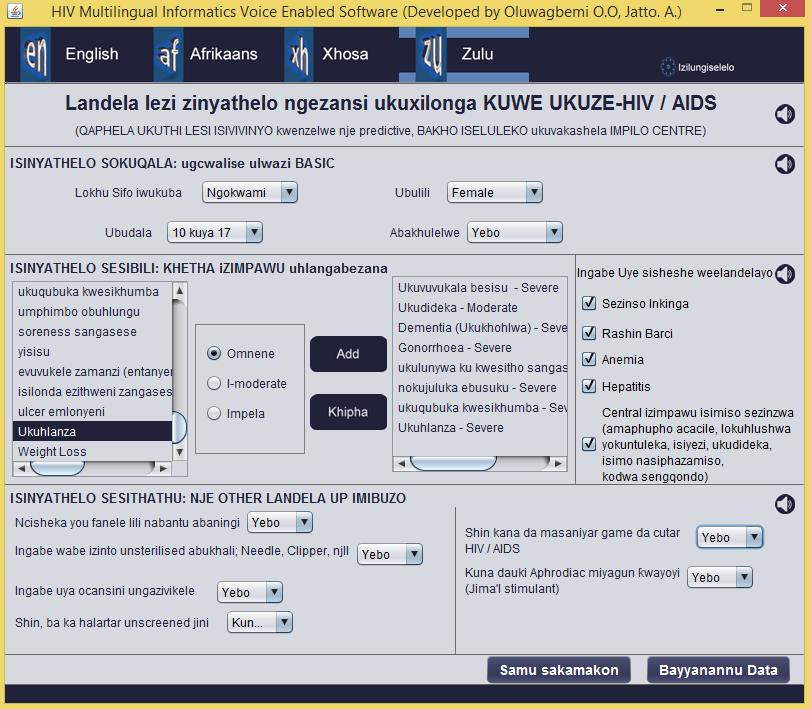
HIV Symptoms keyed-in in Zulu Language

Figure S13. The Graphical User Interface of the HIV Informatics Multi-lingual Voice Enabled Software showing HIV symptoms keyed-in in Zulu language.

Predicted Results in Zulu Language, for selected HIV symptoms


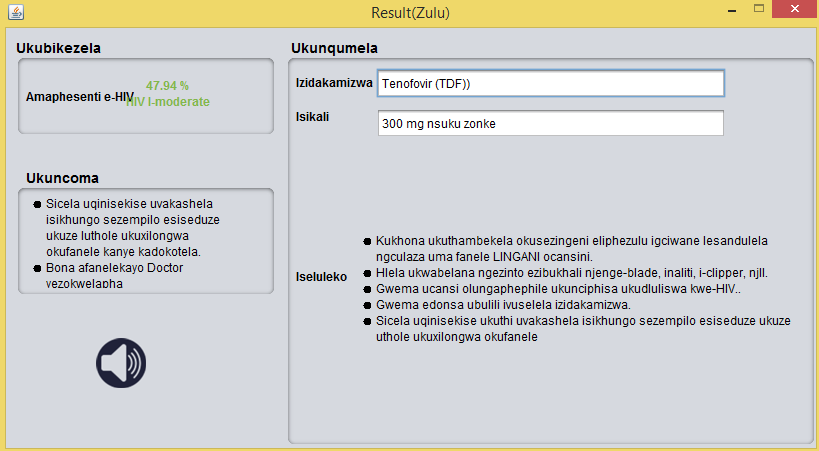


Figure S14. The Graphical User Interface of the HIV Informatics Multi-lingual Voice Enabled Software showing Predicted Results in Zulu Language, for selected HIV symptoms

Appendix S3

Another example to illustrate the implementation of fuzzy rule within the MAVSCOT software is presented here and applied to an experimental sample on HIV diagnoses within MAVSCOT.

The HIV symptoms of a female HIV patient, was keyed into the MAVSCOT software. These HIV symptoms were obtained from scientific literature (See Table S1). The algorithm for the MAVSCOT software was implemented and executed.

Second, the theory of fuzzy rule, fuzzy logic and fuzzy sets was implemented for the HIV symptoms of the female patient.

Third, the predicted diagnosis result from the MAVSCOT software was produced.

The subsequent sections provide a comprehensive description of this process:

We demonstrated this process by considering an example of a female HIV patient. We obtained 24 HIV symptoms (Weight Loss, Vomiting, Ulcer on the Genitals, Swollen Lymph Nodes, Stomach Upset, Soreness of the Vagina, Sexual Dysfunction, Painful Urination, Painful Intercourse, Pain in the Abdomen, Missed periods, Lower Abdominal Pain, Joint Pain, Itching in the Vaginal Area, Heavier or Lighter Periods, Gonorrhea, Forgetfulness, Depression, Dementia, Diarrhea, Body Temperature, Anxiety, Abnormal vaginal discharge, Abdominal swellings ), from scientific literature for the female patient (See Table S1 and Table S13). These HIV symptoms were keyed into the MAVSCOT software.

The MAVSCOT Algorithm was applied and the process is described below:

Step 1: HIV symptoms of the female HIV patient were extracted from medical and scientific literature [See Table S1.]. These HIV symptoms were re-coded into symbols, and tabulated into a new table showing the symbols, HIV symptoms, severity of the symptoms, and rating of the variables and generation of a Triangular fuzzy function values [See Table S14].

Step 2: Table S13 was used to generate the Triangular Fuzzy function values. See Table S13 and Table S14.

Step 3: Sample diagnosis was conducted by inputting the HIV symptoms into the MAVSCOT software under different forms of Fuzzy Rules (Rule 1 to Rule 14) and under different severity, coupled with corresponding possible behavioural lifestyles of patients. All these were captured and specified within the MAVSCOT software.

This process was carried out for the MAVSCOT English software, MAVSCOT Afrikaans software, MAVSCOT Xhosa (IsiXhosa) software, and the MAVSCOT Zulu software.

The predicted results for each of the software module were recorded in the Fuzzy Rule Base table. See Table S12.

Step 4: From Table S12, a new table was generated [See Table S15] depicting the rules that generated non-zero minimum values.

Step 5: The Rules that produced non-minimum zero values for different severity of HIV symptoms was extracted from Table S12.

So from Table S15,

For Mild cases of the HIV symptoms of the female patient, we have that:

Mild **= $\sqrt{R_{2}^{2}+R_{3}^{2}}$ = $\sqrt{0^{2}+0^{2}}$=** $\sqrt{0^{2}=}$0

For the Moderate cases of the HIV symptoms of the female patient, we have that:

Moderate = $\sqrt{R_{1}^{2}+R_{5}^{2}+R_{9}^{2}+R_{13}^{2}+R_{14}^{2}}=$ $\sqrt{0.33^{2}+0.33^{2}+0.33^{2}+0.33^{2}+0.33^{2}}=$ $\sqrt{0.1089+0.1089+0.1089+0.1089+0.1089}=$

$\sqrt{0.5445}=$0.7379

For the Severe cases of the HIV symptoms of the new female patient, we have that

Severe **=** $\sqrt{R_{4}^{2}+R_{6}^{2}+R_{7}^{2}+R_{8}^{2}+R_{10}^{2}+R_{11}^{2}+R_{12}^{2}}=$ $\sqrt{0.67^{2}+0.67^{2}+0.67^{2}+0.67^{2}+0.67^{2}+0.67^{2}+0.67^{2}}=$ $\sqrt{0.4489+0.4489+0.4489+0.4489+0.4489+0.4489+0.4489}=$

$\sqrt{3.1423}=$1.77265 = 1.7727

Step 6: In this step, the Center of Gravity (CoG) Technique was applied and defuzzification process takes place as follows:

Mild **=** $\frac{Mild(valueobtainedinStep5)}{NumberofRules}=$ $\frac{0}{2}=$0

Moderate **=** $\frac{Moderate(valueobtainedinStep5)}{NumberofRules}=$ $\frac{0.7379}{5}=0.1476$

Severe **=** $\frac{Severe(valueobtainedinStep5)}{NumberofRules}=$ $\frac{1.7727}{7}=0.2532$

Step 7: The fuzzy set is defuzzified into precise outputs. Applying the CoG method, defuzzification goes as follows:

Output = $\frac{(0X0.2)+(0.1476X0.45)+(0.2532X0.8)}{0+0.1476+0.2532}=$ $\frac{(0+0.06642+0.20256)}{0.4008}=$ $\frac{0.26898}{0.4008}=$ $\frac{0.27}{0.4}X100=$67.5%

Output = 67.5%

Step 8;

Output $\approx$68% prediction of HIV diagnosis. This MAVSCOT predicted result reveals the possible severity of HIV in the female patient’s body.

See Table S10 to see the MAVSCOT software predicted results. This results reveal that the female patient has been diagnosed with HIV at the severe stage. The patient seems to have entered into the advanced chronic HIV infection stage [See Table S2].
